# Co-designing a culturally appropriate mHealth physical activity intervention for midlife women experiencing menopause in Saudi Arabia: stakeholder recommendations

**DOI:** 10.1186/s12889-026-27624-6

**Published:** 2026-05-27

**Authors:** Ghada AlSwayied, Rachael Frost, Fiona L Hamilton

**Affiliations:** 1 UCL Research Department of Primary Care and Population Health, University College London, Upper 3rd Floor Royal Free Campus, Rowland Hill Street, London, NW3 2PF UK; 2 Department of Community Health Sciences, King Saud University, Riyadh, Saudi Arabia; 3 School of Public and Allied Health, Liverpool John Moores University, Liverpool, UK

**Keywords:** Women, Middle-aged, Midlife, Menopause, Menopausal transition, Perimenopause, Menopausal symptoms, Self-care, Physical activity, Exercise, Behaviour change, Digital interventions, mHealth, Mobile applications, Apps, Culture, Saudi Arabia, Co-design, Participatory research, Stakeholder engagement

## Abstract

**Background:**

Promoting Physical activity (PA) is a public health priority, with regular engagement associated with improved psychosocial and physical outcomes during menopause. Despite these benefits, adherence to PA guidelines among midlife women remains low globally, a pattern particularly pronounced in conservative contexts such as Saudi Arabia. mHealth presents a promising opportunity for supporting PA behaviour change at scale, yet effective engagement requires interventions aligned with women’s needs and preferences. This study aimed to co-generate and prioritise actionable recommendations for a contextually tailored mHealth PA intervention for midlife Saudi women experiencing menopause.

**Methods:**

This study employed an exploratory qualitative design guided by the Generative Co-design Framework for Healthcare Innovation. Online group-based workshops were conducted between May and October 2024. In the first round, four co-ideation workshops were held with homogeneous groups of potential end-users (midlife Saudi women) and professionally diverse stakeholders relevant for intervention development, using storyboards to elicit women’s perspectives of PA-related needs and challenges. The second round involved a cross-sectoral co-prioritisation workshop using a consensus-building matrix. Workshops were audio-recorded, transcribed, and analysed using inductive qualitative content analysis. Stakeholder-generated recommendations were subsequently mapped onto the COM-B model and Behaviour Change Wheel (BCW).

**Results:**

Twenty-three stakeholders participated across five co-design workshops, including midlife Saudi women (*n* = 3), various healthcare providers (HCPs) (*n* = 14), policymakers (*n* = 2), fitness trainers (*n* = 3), and an app developer, each participating in at least one workshop. Agreed priorities included tailored educational modules on menopause and PA, a home-based adaptable exercise library, empathetic messaging framing PA as self-care, progress tracking and feedback, personalisation options, and social and community support features. Cultural tailoring was identified as essential across all components. Stakeholders also recommended an ecosystem approach to implementation, encompassing technical training and intergenerational family digital support to enhance digital skills and inclusion, integration with existing health systems and HCP endorsement, and cross-sectoral partnerships to support uptake and sustainability.

**Conclusion:**

This study represents one of the first applications of a structured participatory co-design approach to digital health intervention development within the Saudi Arabian context. The co-design process proved feasible and acceptable and generated culturally grounded insights for a population that remains underrepresented in health research. The findings provide a foundation and practical direction for intervention content, design features, and implementation strategies aligned with women’s lived experiences and sociocultural context. The necessary next steps are prototype development and feasibility testing.

**Supplementary Information:**

The online version contains supplementary material available at 10.1186/s12889-026-27624-6.

## Introduction

Physical activity (PA) is widely recognised as a public health priority, playing a key role in the prevention of non-communicable diseases (NCDs), improving mental health and well-being, and enhancing overall quality of life [1]. Among midlife women undergoing the menopausal transition, engaging in moderate levels of PA and exercise has been associated with reduced psychosocial and physical menopause-related symptoms [2–5]. PA has also been viewed positively as a potential strategy for managing some menopausal symptoms and for supporting long-term health benefits for healthy ageing [6, 7]. Despite these benefits, adherence to PA guidelines remains low among women in this age group, highlighting the need for targeted interventions. For example, in England, 38% of women aged 45–54 engage in less than the recommended 150 min of moderate activity per week, and 60% perform strength training less than twice weekly [7].

Menopause-related physical and psychological symptoms, including fatigue, musculoskeletal pain, weight gain, hot flushes, and mood changes, can reduce motivation and perceived capability to engage in PA [8]. These barriers are often compounded by body image concerns and stigma surrounding ageing and menopause, particularly in public exercise settings [8–10], as well as time constraints arising from work, family, and caregiving responsibilities [9, 11–13]. These challenges to PA during menopause may be particularly pronounced in conservative sociocultural settings such as Saudi Arabia [14]. In the Saudi context, cultural norms and environmental factors have created distinct barriers to PA and exercise among women, including traditional gender role expectations, modesty norms, limited access to women-only exercise facilities, and inadequate public spaces for PA [15, 16]. According to the Saudi Arabian National Survey on PA [17], only 46% of adult women meet recommended PA thresholds, compared with 64% of men, reflecting an 18% gender gap. Participation rates for both genders decline notably during midlife [17], a period coinciding with the menopausal transition, suggesting that Saudi women at this life stage could face particular challenges for PA engagement.

​The use of technology, particularly mobile health (mHealth) interventions, offers a potentially promising approach for addressing accessibility and scalability challenges. In Saudi Arabia, internet use is nearly universal, with mobile phones accounting for 98.9% of online access [18], suggesting a strong infrastructure to support mHealth solutions. Research demonstrates that mHealth tools can support PA behaviour change, with reviews suggest modest to moderate increases in PA across various adult populations [19, 20]. Among older adults, an umbrella review reported moderate effects of mHealth interventions such as apps, wearables and SMS on step counts and small effects on moderate-to-vigorous PA, with no significant differences across intervention types [21]. For midlife women, a systematic review suggests that mHealth PA interventions may support small to moderate increases in moderate to vigorous PA, and may lead to positive improvements in some menopausal symptoms such as anxiety, sleep, and menopause-related quality of life [22].

However, interventions intended to effectively support PA engagement among midlife Saudi women experiencing menopause require a context-sensitive understanding of women’s needs, preferences, and lived experiences. Narrative review evidence suggests that cultural tailoring in digital health interventions is more likely to be effective when culture is considered alongside other contextual influences of the target population, e.g., social structures, economic conditions and existing health systems, and when diverse stakeholders are involved to generate practical insights throughout the design and evaluation [23].

Co-design, defined as a participatory approach involving relevant stakeholders as active collaborators, has emerged as a valuable methodology for health intervention development [24, 25]. This participatory approach ensures that the interventions and services developed would likely reflect the real needs and experiences of the target population [26]. Evidence suggests that integrating co-design into health intervention development improves their user-centeredness, acceptability, relevance, and alignment with the needs of the individuals they aim to serve [27–32]. Thus, involving potential end-users and other relevant stakeholders can help create interventions that are both evidence-based and contextually appropriate [31].

The present study aimed to gather stakeholders’ insights to build on findings from our previous in-depth interviews with midlife Saudi women experiencing menopause [33] and to co-design actionable recommendations for more acceptable and feasible intervention strategies. These recommendations were intended to inform the development of an optimised mHealth PA intervention tailored for midlife women undergoing menopause in Saudi Arabia.

## Methods

### Study design

This study utilised an exploratory qualitative design, incorporating participatory and stakeholder engagement approaches, guided by a generative co-design process [34], consisting of three main stages: pre-design, co-design, and post-design. This co-design methodology facilitates knowledge sharing among stakeholders, who actively engage in a collective creative process aimed at developing innovative solutions, such as digital health interventions. By fostering collaboration, this approach helps create an environment where health innovations that are context-specific, relevant, and acceptable can be developed [34].

This study is part of research that was guided by Public and Patient Involvement (PPI) members [33], specifically two middle-aged Saudi women experiencing menopause. The PPI members offered valuable input on the study objectives, the co-design activities, workshop format, and selection of relevant stakeholders.

A total of five workshops with different stakeholders were conducted. Due to resource limitations and time constraints, these group-based sessions were held online via Microsoft Teams. The two PPI members also recommended the online format to maintain convenience and ensure privacy for menopausal Saudi women. The consolidated criteria for reporting the 32-item checklist of qualitative studies guided the reporting of this study [35].

### Phase 1: pre-design

In the pre-design stage, the lead researcher (GS) conducted a comprehensive review and synthesis of the key themes identified from a prior contextual inquiry of 29 in-depth interviews with midlife Saudi women, of which one paper has been published [33]. The qualitative interview study explored midlife Saudi women’s attitudes and experiences of menopause [33], as well as their barriers and facilitators to PA participation during this life stage, which will be reported in a separate paper. The interviews also explored midlife Saudi women’s perceptions and experiences of using mHealth and smartphone-based interventions to support PA participation. Findings from the reflexive thematic analysis of these interviews informed the present co-design process, after which workshop materials were developed and stakeholders identified and recruited.

#### Workshop material development

We developed a workshop topic guide (supplementary file 1) informed by the prior interview findings, structured around the Behaviour Change Wheel (BCW) and COM-B theoretical frameworks [36, 37] and refined based on feedback from senior qualitative researchers (FH and RF) and PPI members. Key constructs explored across the workshops included but not limited to appropriate intervention types and functions, behavioural change strategies, menopause relevance, cultural alignment, design prioritisation, feasibility, delivery modes, accessibility and digital inclusion, and system integration. We also prepared informational materials, and presentation slides to introduce potential stakeholders to the co-design workshops’ aims and structure, facilitating informed participation. PPI feedback was gathered on the Arabic versions of the study materials, including the participant information sheet, informed consent form, study posters, workshop topic guide, presentation slides, and storyboards.

#### Stakeholder identification and participation

Since considering diverse perspectives when forming co-design activities is seen crucial to ensuring inclusivity in decision-making processes [38], the research team included two key groups of stakeholders: midlife Saudi women with menopause lived experience (referred as potential end-users), and multidisciplinary professionals, including healthcare providers (HCPs), qualified fitness trainers, policymakers and technology developers and designers. The recruitment process for the co-design workshops was conducted between March-September 2024.

Midlife Saudi women who had previously participated in our earlier, qualitative interview-based study (*n* = 29) were invited to participate. Building on the rapport established [33], these women were re-contacted and encouraged to continue their involvement as end-user stakeholders. The purposive sampling approach and eligibility criteria followed were comprehensively reported earlier [33]. Briefly, we included middle-aged women aged between 40 and 64 years, undergoing menopause (either natural or sudden menopause due to e.g. cancer treatments or surgeries); based in Saudi Arabia; not meeting the recommended PA guidelines [39]; and with internet access to facilitate participation in online workshops. Owning a smartphone was not a criterion for participation. We targeted women who self-reported as inactive or insufficiently active to ensure that the co-designed recommendations address the needs and barriers of those most in need of PA support. As a token of appreciation for their time and contribution, each participant was offered a £20 (100 SAR) voucher upon completion.

A purposive sampling approach was employed to recruit a multidisciplinary group of Saudi-based professionals, including HCPs (such as general practitioners, gynaecologists, physiotherapists, psychologists, and health educators), certified fitness trainers, health policymakers, and technology experts. Professional stakeholders were identified through established personal networks and LinkedIn connections, and through social media posts using a separate invitation poster specifically designed for professional audiences. Professional stakeholders did not receive compensation but in recognition of their valuable time and contributions each of them received a formal thank-you letter acknowledging their participation in the co-design workshops.

Once a potential stakeholder expressed interest in participating in the co-design workshops, an information pack was sent to them to provide further details about the co-design study prior to completing the electronic informed consent and demographic questionnaire.

#### Facilitators

The workshops were facilitated by the lead researcher (GS), a Saudi PhD researcher with experience and training in qualitative and co-design research, who guided discussions and ensured that participants remained focused on the problem areas being addressed, while fostering an inclusive environment where all participants could contribute equally. The second facilitator, FD, a female Saudi health education specialist experienced in co-facilitating focus groups, was responsible for taking notes, supporting communication, and assisting with the completion of co-design activities to ensure all contributions were accurately documented. Before the workshops, the second facilitator (FD) was briefed on the study’s aims and the expected role, ensuring alignment in their approach. Following each workshop, the facilitators met to discuss key takeaways and refine subsequent sessions based on emerging insights. Together, they produced a brief document summarising the overarching narrative from each workshop. Any discrepancies or differing perspectives were resolved collaboratively through online discussions.

#### Intended workshop deliverables

Through iterative discussions and feedback cycles, the co-design workshops produced actionable recommendations for key intervention strategies, messaging, and design features. These outputs informed the preliminary conceptual development of future optimised mHealth PA interventions targeting menopausal women in Saudi Arabia.

### Phase 2: co-design

**Fig. 1:**
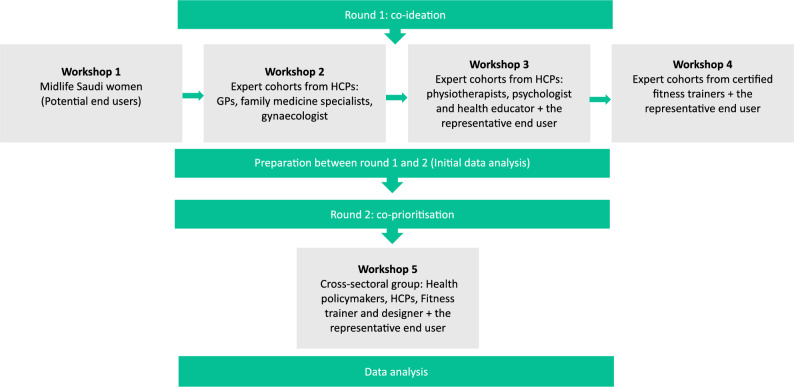
Flow of co-design workshop across two rounds and the contributions of diverse stakeholder groups

The co-design phase marks the stage where stakeholders and facilitators actively engaged in co-design activities. According to the generative co-design approach, this phase consists of framing the issue, conducting generative design work, and sharing ideas [34]. In our workshops, we observed that these steps were interrelated and unfolded dynamically, with participants seamlessly transitioning between these within a single session. Our co-design workshop flow structure (Fig. 1) integrated two rounds of the co-design phase, highlighting their interconnected nature and the ongoing iterative development throughout the entire co-design process.

#### Data collection and workshop flow structure

Workshops were held between May and October 2024 via Microsoft Teams by the primary investigator (GS), and each lasted between 1.5 and 2 h. All the five co-design workshops were conducted in Arabic, except for one session with HCPs, which was held in English to engage with non-Saudi, non-Arabic-speaking clinicians and ensure their perspectives were also captured.

Each workshop commenced with an introductory meet-and-greet to set a welcoming tone. Ice-breaker activities were included to ease participants into the discussions, fostering open communication. Ground rules were collectively established, promoting principles of active listening, mutual respect, confidentiality, and equal opportunity for participation. This collaborative approach aimed to create a safe, judgment-free space where stakeholders felt empowered to share their insights and ideas freely. Recognising the possibility of fatigue during workshops, we built in regular breaks between activities and encouraged participants to stay hydrated and take refreshments. Although no fixed guidelines exist for conducting co-design activities, we adopted a homogenous and phased structure previously employed in community settings [31].

#### Round one: co-ideation with like-minded groups

Workshops 1–4 aimed to co-ideate with like-minded stakeholders. This homogenous group approach enabled co-facilitators to build upon insights and feedback from one session to another, creating a progressively evolving co-design process. Each workshop began with a *sensitising* activity, defined as “*preparing participants for the design task and triggering reflections on the topic through activities such as presentations and thought-provoking questions*” [40]. Participants were sensitised through a brief presentation summarising key themes (barriers and needs) identified in the preceding in-depth interviews, along with insights from previous workshops (based on facilitator notes). This visualisation was intended to foster open and reflective discussion.

Stakeholders were encouraged to share, debate, and reflect on their lived experiences and/or professional insights, particularly concerning the contextual requirements for promoting PA participation among menopausal women in Saudi Arabia. We then facilitated creativity and solution generation through a collaborative co-design activity (ideation phase of co-design).

**Fig. 2:**
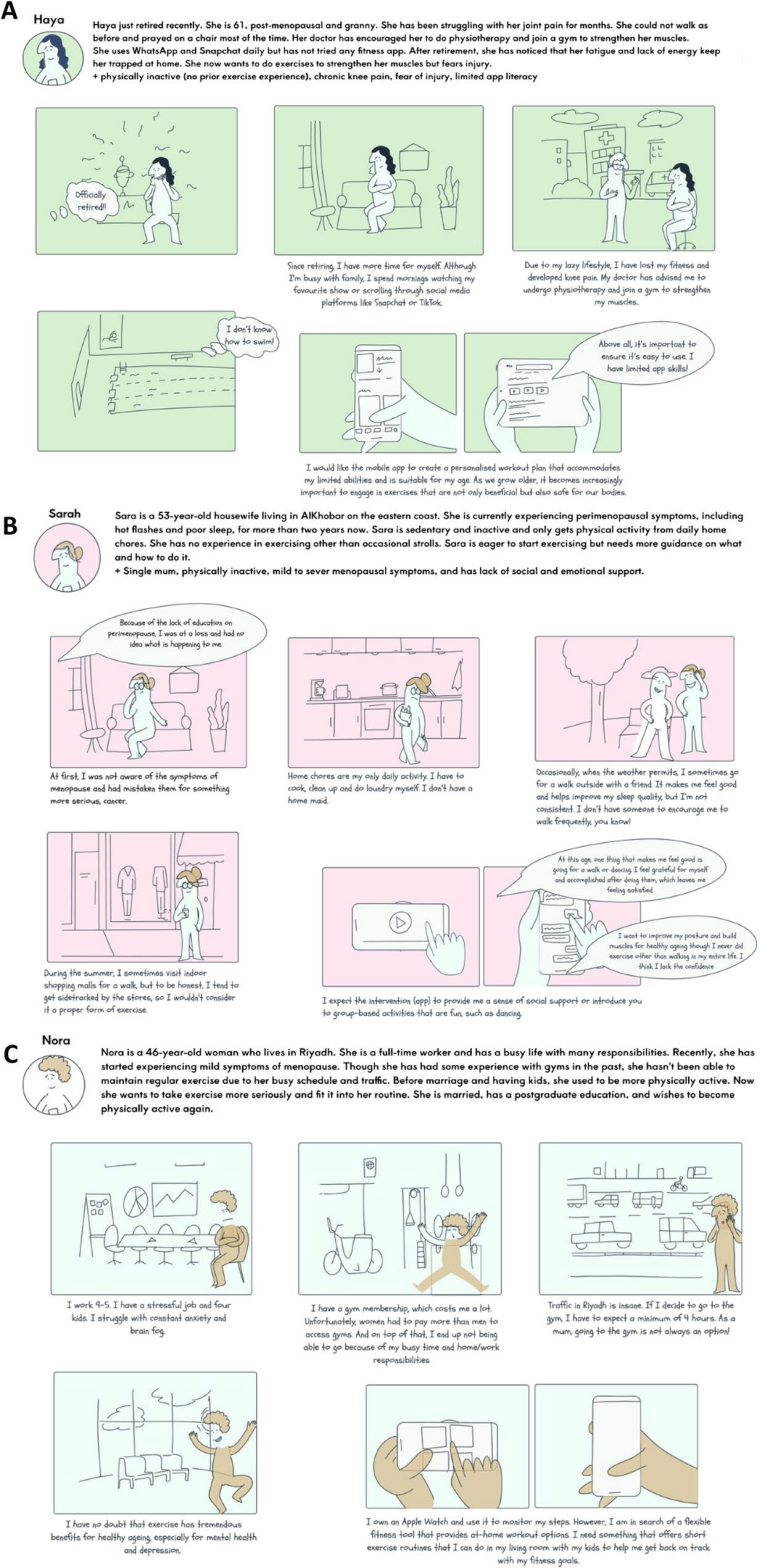
**A** Storyboard vignettes used in the co-design workshops 1–4. **B** Storyboard vignettes used in the co-design workshops 1–4. **C** Storyboard vignettes used in the co-design workshops 1–4

To actively engage stakeholders, build empathy and ground the discussion in real-world experiences and contexts, we utilised adapted real-life, case scenarios based on the previous interview study, illustrated through storyboards (Fig. 2A-C). The personas in the storyboards: Haya, Sarah, and Nora (pseudonyms) embodied shared challenges and opportunities faced by menopausal women in Saudi Arabia.

Stakeholders then participated in think-aloud brainstorming sessions aimed at generating innovative ideas. These sessions focused on imagining approaches for a mobile-based intervention to support the fictional personas in increasing their participation in PA. Facilitators guided discussions by posing questions such as: “How should the mobile PA intervention reflect the menopause experience to ensure relevance for persona X?”, “What strategies should the intervention use to address the challenges persona X faces?”, and “What features are needed, and how might the personas interact with these features?”.

The ideas generated were then discussed, focusing on translating them into actionable recommendations to specifically address the challenges outlined in the scenarios. This process highlighted key elements of emphasis and importance, forming a foundation for tailoring the PA intervention to meet the needs of the personas.

#### Round two: co-prioritisation with a cross-sectoral group

In Workshop 5, we convened a diverse group of stakeholders, including policymakers from the Saudi Ministry of Health (MOH), an app designer, along with a subset of stakeholders from previous workshops (1–4): a general practitioner (GP), a fitness trainer, and a representative from midlife Saudi women. The objective was to collaboratively refine and prioritise the proposed intervention directions that had been generated during co-ideation workshops.

**Fig. 3:**
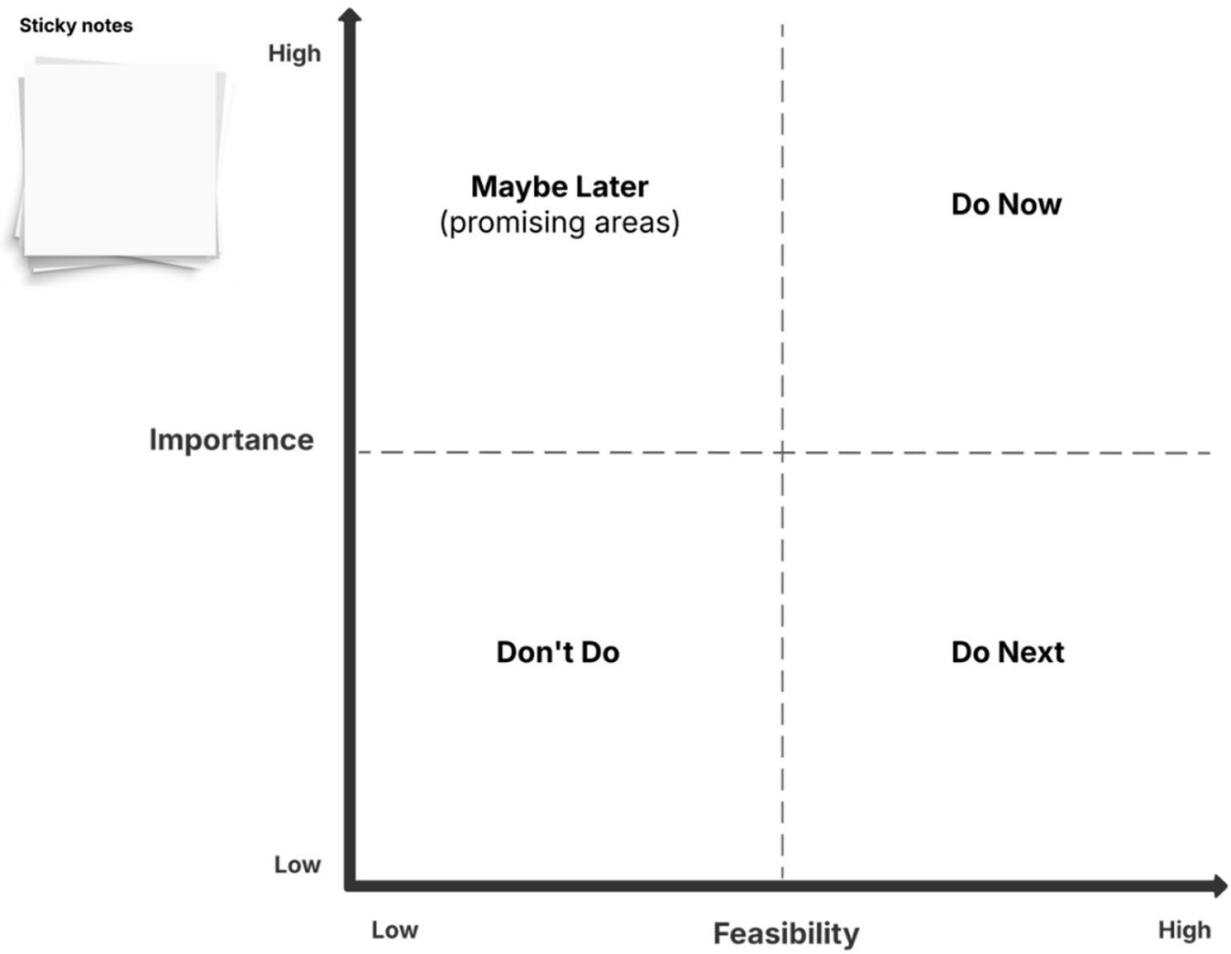
Adaptable 2 × 2 prioritisation matrix used in Workshop 5, shared with stakeholders via Lucidspark Software (https://lucidspark.com)

Prior to Workshop 5, the facilitators prepared a list of the generated ideas from earlier workshops 1–4. Workshop 5 aimed to reach consensus on the proposed strategies (evaluate phase of co-design). To facilitate this, we presented an adapted 2 × 2 priority matrix on a shared slide in order to categorise each item of the list generated based on two criteria: *importance*/*impact* which indicate how crucial the suggested strategy (i.e., app component) is to addressing menopausal Saudi women’s needs and improving their participation in PA, and *feasibility* which indicates how easily the suggested strategy can be developed, implemented, and maintained considering time, cost, and resources. We asked stakeholders to collaboratively rank them within one of the designated quadrants: high-impact, high-feasibility (Do Now); low/moderate-impact, high-feasibility (Do Next); high-impact, low/moderate-feasibility (Promising, Maybe Later); and low-impact, low-feasibility (Do Not Do) (Fig. 3).

To facilitate discussion, stakeholders were encouraged to evaluate the proposed intervention strategies within the Saudi context using the APEASE criteria: Affordability, Practicability, Effectiveness/cost-effectiveness, Acceptability, Side effects/safety, and Equity [41]. The APEASE criteria are typically used within the Behaviour Change Wheel (BCW) to assess the feasibility of implementing a new intervention within a specific context, as described by Michie et al. [41].

During Workshop 5, stakeholders explored the feasibility of implementing the proposed strategies for designing, delivering, and integrating within the Saudi Arabian context. Discussions addressed logistical challenges, potential methods for testing, and strategies for achieving widespread implementation, with an emphasis on ensuring long-term intervention sustainability.

#### Representation of end-user voices

To ensure the voices of midlife Saudi women experiencing menopause were represented throughout the co-design process, the research team invited an end-user representative (Stakeholder 1: a perimenopausal Saudi woman) to participate. This representative had participated in both the initial interview study and Workshop 1 and expressed a keen interest in remaining involved. With her consent, her real, first name was acknowledged in the research to authentically amplify her voice and perspective. Contributions from this individual were incorporated throughout Workshops 3, 4, and 5, offering valuable insights to ensure the outcomes aligned with the lived experiences of menopausal Saudi women. The representative was unable to attend Workshop 2, which was delivered entirely in English to incorporate perspectives from non-Arabic speaking HCPs. Ongoing engagement was supported through video calls and WhatsApp messaging.

#### Rigour and reflexivity

Efforts were made to avoid influencing stakeholders’ perspectives at the outset of each co-ideation workshop. The topic guides, prompts, and storyboards used were intentionally kept generic, without introducing specific app functionalities or design features, to prevent biasing stakeholders’ input. Instead, ideas emerged organically through the co-design activities in which stakeholders actively participated. This approach aligns with best practices in co-design methodology [27].

Although the co-ideation workshops 1–4 followed a similar structure, they were intentionally designed to be flexible. This flexibility allowed stakeholders to guide the discussions and prioritise the aspects of the proposed solutions that they deemed most relevant, reflecting the reflexivity and participatory principles central to co-design approaches [27].

Facilitators remained neutral, refraining from offering personal opinions or reactions during discussions, with reflexive notes after each workshop capturing any potential instances where facilitator input might have influenced discussions. Regular debriefing sessions among the research team helped maintain awareness of these dynamics throughout the process. Despite these precautions, we acknowledge that the presence of facilitators and the nature of questioning may still have subtly shaped the discussions, as is inherent in constructivist qualitative research methodologies [42]. This limitation is common in participatory research but was mitigated through our transparent documentation.

The lead researcher’s (GS) position as a Saudi female healthcare professional and PhD researcher with background knowledge of both menopause and PA interventions was continuously acknowledged throughout the research process. This positionality created both advantages in terms of cultural understanding, richer discussion, and rapport-building with participants, particularly from midlife Saudi women (potential end-users), while also necessitating careful attention to avoid imposing preconceived ideas about appropriate intervention features. Our reflexive approach involved critically examining how our professional backgrounds and cultural identities might influence the co-design process, seeking to leverage these perspectives as resources while privileging the expertise of stakeholders regarding their lived experiences and contextual realities.

### Phase 3: post-co-design

The post-co-design phase encompasses data analysis and requirements translation [34]. These steps occurred simultaneously and iteratively, with data analysis commencing early in the process and directly informing Round 2 of Workshop 5. This iterative analytical process continued beyond Workshop 5, ensuring a dynamic refinement of stakeholders’ priorities.

An inductive qualitative content analysis (QCA) approach was used to analyse the workshop findings. In alignment with the participatory nature of co-design [27], and following thoughtful discussions with the research team, QCA was chosen for its ability to identify descriptive themes while remaining close to stakeholders’ voices and language [43, 44], in order to treat them as co-design partners rather than as passive subjects. Its pragmatic and structured nature makes it especially suited to applied contexts such as intervention development [45, 46].

All workshops were audio-recorded, transcribed verbatim, anonymised, and managed using Microsoft Excel (Microsoft Corporation, Redmond, WA). Identifying information was removed from transcripts to ensure confidentiality. GS conducted qualitative synthesis of the workshop data including transcripts, facilitator notes, and reflective summaries compiled at the end of each session. Data from each workshop were tabulated and triangulated to identify convergent and divergent insights and ideas across sessions. Recurring codes were grouped across workshops and refined into meaning-based patterns (themes). These themes formed the basis for the ‘proposed intervention design strategies’, capturing the core insights of diverse stakeholder groups. Illustrative excerpts from participants were integrated to contextualise and substantiate the final recommendations. The data synthesis and consensus were verified by senior researchers (FH and RF).

The co-design process was inherently iterative, involving a continuous cycle of identifying areas of agreement, disagreement, and emerging considerations. Insights evolved progressively through ongoing discussions and reflection. The first four workshops were conducted sequentially, with each session building on the previous one. At the start of each workshop, an overview of key insights identified from the preceding session’s facilitator notes was shared to inform discussions.

Following the completion of the four workshops (round 1), a comprehensive synthesis of the transcripts was conducted to capture overarching themes. Abstract ideas generated from stakeholder input were organised and synthesised into potential intervention directions, translating stakeholder insights into clearly defined intervention design strategies. These proposed collective strategies were then re-circulated to stakeholders during Workshop 5 for co-prioritisation and final reflection, enabling participants to engage with the consolidated findings and further refine the actionable recommendations for intervention design. The outputs of this final workshop were then synthesised to produce the final set of intervention design recommendations.

Subsequently, the co-design findings were mapped onto the COM-B model, the TDF domains, and the BCW intervention functions to identify potential behavioural mechanisms underlying stakeholder co-designed recommendations for future intervention development [36]. We used a sequential approach in which behaviour change theory was introduced after the co-design activities as a secondary, interpretive layer to contextualise stakeholder-generated recommendations, aiming to improve conceptual clarity and replicability while preserving the participatory nature of the co-design process. The lead researcher (GS) conducted the mapping, and two senior researchers (FH and RF) reviewed the outputs independently. Any discrepancies were discussed until consensus was reached.

### Ethical considerations

Ethical approval for this study was granted by the University College London Ethics Review Committee (London, UK) as an extension to the qualitative in-depth interview study (reference number 23,817/001). Local ethical approval was also obtained from King Saud University, Riyadh, Saudi Arabia (ID no: 22/0477/IRB). Prior to participating, all participants provided written informed consent after reviewing the study information sheet. Alongside the consent form, socio-demographic information was collected online via Microsoft Forms before the workshop day. While some experts among the stakeholders were proficient in English, all participants were given the choice to conduct the sessions in Arabic (their first language) or English. Consent materials, including the participant information sheet and informed consent forms, were translated into plain Arabic. With the participants' permission, workshops were audio-recorded using Microsoft Teams, and verbal as well as written confirmation of their consent was obtained.

All participating stakeholders voluntarily agreed to engage in the co-design workshops and activities, with ethical considerations strictly adhered to throughout the process. Confidentiality was maintained at every stage of the study. Participants were reminded of their right to withdraw from the study at any time. Due to the nature of group discussions, participants were informed that it might not be possible to remove data already shared if they decided to withdraw after the workshop, but their data will be excluded from the final transcripts and analysis. In practice, no participants withdrew following their involvement in the workshops.

## Results

### Participant characteristics

Twenty-three participants were involved across five co-design workshops, comprising potential end-users and key professional stakeholders. Geographically, participants were based across key regions of Saudi Arabia: Central (Riyadh), Eastern (Al-Khobar and Dammam), and Western (Jeddah and Makkah) (see Table 1).

**Table 1:** Demographic data of the participating stakeholders (*N*= 23)

**End user co-designer** ^**a**^	**Menopause stage**	**Marital status**	**Occupation**	**Educational qualification**	**Geographical region**
Stakeholder 1	Perimenopausal ^b^	Married	Housewife	University	Dammam
Stakeholder 2	Perimenopausal ^b^	Single	Part-time work	Secondary	Jeddah
Stakeholder 3	Post-menopausal ^c^	Married	Retired	Postgraduate	Riyadh

**Professional co-designer**	**Gender**	**Nationality**	**Occupation**	**Work experience**	**Geographical region**
Stakeholder 4	Female	Non-Saudi	Gynaecologist, University Educational Hospital	10 yrs	AlKhobar
Stakeholder 5	Female	Saudi	Family Medicine Doctor, Women's Health Clinics, Ministry of Health	8 yrs	Riyadh
Stakeholder 6	Female	Saudi	Family Medicine Doctor, University Educational Hospital	9 yrs	AlKhobar
Stakeholder 7	Female	Non-Saudi	Family Medicine Doctor, Women's Health Clinics, Ministry of Health	7 yrs	Dammam
Stakeholder 8	Female	Saudi	Family Medicine Doctor, Ministry of Health	6 yrs	Riyadh
Stakeholder 9	Female	Saudi	Physiotherapist, Military hospital	9 yrs	Riyadh
Stakeholder 10	Female	Saudi	General Practitioner, Military hospital	7 yrs	AlKhobar
Stakeholder 11	Female	Non-Saudi	General Practitioner, Ministry of Health	8 yrs	Jeddah
Stakeholder 12	Female	Saudi	Clinical Psychologist, Behavioural therapy, Private Mental Health Medical Centre	5 yrs	Riyadh
Stakeholder 13	Female	Non-Saudi	General Practitioner, University Educational Hospital	10 yrs	AlKhobar
Stakeholder 14	Female	Non-Saudi	Family Medicine Doctor, University Hospital	7 yrs	Jeddah
Stakeholder 15	Female	Saudi	Occupational Therapist, Ministry of Health Tertiary Hospital	9 yrs	Riyadh
Stakeholder 16	Female	Saudi	Occupational Therapist, Military Medical hospital	10 yrs	AlKhobar
Stakeholder 17	Female	Saudi	Health Education Specialist, Non-profit Obesity Prevention Organisation	2 yrs	Riyadh
Stakeholder 18	Female	Saudi	Physical Fitness trainer, Private Gym	6 yrs	Riyadh
Stakeholder 19	Female	Saudi	Physical Fitness trainer, Private Gym	3 yrs	Jeddah
Stakeholder 20	Female	Saudi	Physical Fitness trainer, Private Gym	3 yrs	Makkah
Stakeholder 21	Female	Saudi	Policymaker, Ministry of Health	12 yrs	Riyadh
Stakeholder 22	Male	Saudi	Policymaker, Ministry of Health Primary Care	11 yrs	AlKhobar
Stakeholder 23	Male	Non-Saudi	App designer, University IT programmer and Founder of a digital company	8 yrs	Riyadh

^a^_Public members from midlife Saudi women (potential end-users)_

^b^_Perimenopausal, self-reported as currently experience irregular period cycle_

^c^_Post-menopausal, self-reported as no periods for 12 months or more_

For the end-user group, all previously interviewed midlife Saudi women were contacted (*n* = 29). Seven women expressed interest and provided consent. Two did not respond to scheduling communications, and five confirmed attendance the day before the workshop. Of these, two were unable to attend due to last-minute conflicts, resulting in three end-users participating. A total of 35 professionals were invited to participate. Of these, 28 expressed interest and provided consent, and 23 responded to workshop scheduling communications. Twenty professionals actively participated in at least one workshop, while three withdrew prior to the workshops due to unforeseen circumstances, including a gynaecologist, a psychologist, and a fitness trainer.

Workshop 1 involved three end-user participants, all of whom were physically inactive or insufficiently active. Two women self-reported as perimenopausal, reporting an irregular menstrual cycle, while one identified as postmenopausal, defined as 12 or more consecutive months without menstruation. Participated women varied in marital status, educational level, and employment status. Workshops 2–5 involved 20 professionals, the majority of whom were female, with only two male professionals, a policymaker and an app designer. HCPs represented a range of specialisms, including general practice, family medicine, gynaecology, physiotherapy, occupational therapy, psychology, and health education (see Table 1).

### Co-design findings

Through collaborative discussions with stakeholders, the co-design workshops provided comprehensive understanding of key PA needs of midlife Saudi women and generated a range of proposed strategies for tailoring mHealth interventions to promote PA during menopause. Table 2 provides an overview of all proposed strategies discussed throughout the co-design workshops.

**Table 2:** Summary of co-designed intervention strategies and implementation priorities

Raw stakeholder insights	Co-created intervention strategies	Suggested features	Targeted SEM levels	APEASE criteria evaluation	Priority
• Low PA literacy amongst middle-aged Saudi women • Misconceptions esp. about strength training • Knowledge gaps about menopause-PA connection • Menopause health support	Tailored educational modules on PA and menopause	• Bite-sized audiovisual modules • Myth-busting content • Interactive quizzes and learning progress trackers	Individual, Institutional	High-impact, highly feasible A: Low cost to develop P: Straightforward implementation E: High for knowledge enhancement A: High for addressing educational needs S: Minimal risk E: Requires health literacy considerations	Do Now
• Physical challenges (mobility limitations, menopause-related joint pain, fatigue) • Confidence barriers and fear of injury • Preference for accessible home-based activities • Need for culturally appropriate private spaces for exercise • Lack of reliable Arabic exercise resources • Affordable compared to gyms	Adaptable yet structured exercise library	• Low impact, modified exercise forms • Step-by-step tutorials with safety guidance • Adjustable playback options	Individual, Institutional, Physical Environment	High-impact, highly feasible A: Cost-effective and scalable with on-demand content P: Practical for home use E: High for skill-building A: High for addressing varied fitness levels and capacities S: Minimal with safety emphasis E: Supports users with mobility limitations	Do Now
• Weight-loss messaging less effective • Preference for self-care framing of PA • Need for menopause-relevant, age-relevant approaches	Empathy-driven messaging	• Local success stories • Empathy-driven (positive) narratives • Self-discovery tools focus on self-care approach	Individual, Societal	High-impact, highly feasible A: Affordable with behavioural support features P: Easy to implement E: Effective for sustaining PA through empathetic encouragement A: Highly acceptable for emotional engagement S: Minimal risks with cultural alignment E: Equitable across diverse menopausal women	Do Now
• Celebration of progress over outcomes • Reinforce holistic benefits of exercise beyond physical transformation. • Motivation from immediate benefits (e.g., energy boosts, mood improvements, pain, sleep quality) • Need for culturally relevant affirmations.	Progress tracking and feedback mechanisms	• Visual progress tracker • Achievement tracking with positive reinforcement • Milestone celebrations with badges • Reflective prompts and reminders e.g., “*I feel energised*”, “*my Ruku and Sujood postures have Improved!”*	Individual	High-impact, highly feasible A: Affordable with simple front-end elements P: Easy to implement with minimal technical demands; can be automated E: Effective for sustaining PA through positive reinforcement; inclusion of spiritual cues may enhance contextual relevance and effectiveness A: Highly acceptable due to emotional resonance S: Minimal risks; focusing on progress not perfection. E: Equitable and adaptable across diverse users	Do Now
• Wide variation in fitness levels and capabilities • Menopausal symptoms vary and fluctuate, new health conditions among midlife women • Need for adaptive approaches	Personalisation and customisation	• Customisable plans based on women’s individual needs • Periodic self-assessment • Adaptive progression system • Filtering tools for exercise library	Individual	High-impact, highly feasible A: Affordable using user data P: Practical for basic features E: High for addressing individual needs A: High for tailored experiences S: Minimal with proper design E: Accommodates diverse users	Do Now
• Value of peer support and accountability • Privacy concerns requiring sensitive approaches	Social and community support	• Women-only forums • Anonymous participation options • Moderated group challenges • Virtual exercise matching buddies	Interpersonal, Societal	Moderate-impact, highly feasible A: Affordable basic features P: Requires moderation E: Moderate for engagement A: High as women-only space S: Moderate risks without moderation E: Connects similar fitness levels and lived experiences	Do Now
• Lack of cultural encouragement for PA in midlife/ menopause • Importance of religious alignment • Need for Saudi cultural context integration	Culturally tailored platform	• Arabic content • Modest visuals • Integration with cultural practices (dance) • Religious practice alignment (e.g., Salah)	Interpersonal, Institutional, Societal	High-impact, highly feasible A: Affordable localisation P: Practical implementation E: High for cultural engagement A: High respect for cultural norms S: Minimal risks E: Ensures cultural inclusivity	Do Now
• Lifestyle resources beyond PA (e.g., meditation, sleep hygiene, nutrition advice, hot flash management)	Lifestyle tips	• Supplementary modules	Individual	Moderate-impact, highly feasible A: Low-cost integration of lifestyle content P: Easy implementation as optional modules without disrupting core PA functions E: Moderately effective - additional guidance can enhance PA adherence A: Acceptable as women value holistic menopause health support S: Minimal risks if expert-led E: Equitable with considerations for literacy, language, and cultural relevance	Do Next
• Need for dynamic, personalised support • Interest in real-time adaptations	AI-adaptive recommendations	• AI adaptation based on symptoms • Dynamic exercise recommendations	Individual	Moderate-impact, less feasible A: Moderately affordable but requires advanced technology P: Less practical due to safety concerns and complexity E: Moderately effective with potential A: Acceptable with expert reservations about reliability S: Potential risks if inaccurate guidance E: Less equitable for those with low digital literacy; barriers noted for middle-aged Saudi women navigating AI tools	Promising, maybe later
• Credibility of Saudi health professional • Medical guidance and support • Menopause: under-discussed in clinical settings, cultural stigma and privacy concerns barriers	Expert tele-consultations	• Professional consultation options	Individual, Interpersonal Institutional	High-impact, less feasible A: High-cost requiring investment in trained professionals P: Less practical due to resource demands E: Highly effective for personalised advice A: Acceptable with female professionals S: Minimal risks with qualified experts E: Less equitable due to limited accessibility; women prefer multiple sessions. Alternative: provide information on accessing menopause care in Saudi Arabia	Promising, maybe later
• Some interest in providing basic equipment • Targeting gym cost issues	Equipment considerations	• Exercise home-kit	Individual, Institutional	Less-impact, less feasible A: High cost P: Logistical challenges E: Low to moderate, not main barrier A: Acceptable being free of charge S: Minimal with guidance E: Equitable if distributed in a way ensuring those most in need or remote areas can benefit	Don’t Do

SEM: Socioecological model. APEASE criteria: Affordability, Practicability, Effectiveness, Acceptability, Side effects/safety, Equity. Priority ratings indicate recommendation for implementation: “Do Now” (immediate), “Do Next” (secondary), “Promising, Maybe Later” (future consideration), and “Don’t Do” (not recommended)

During the prioritisation activity in Workshop 5, stakeholders assessed all proposed strategies against the APEASE criteria [36] and prioritised seven concrete design recommendations from the broader set outlined in Table 1 to form the foundation of a tailored menopause-specific PA mHealth intervention. These priorities appear to address knowledge gaps, motivation and user engagement barriers, and sociocultural considerations essential for promoting PA among midlife Saudi women experiencing menopause. Although stakeholders during the prioritisation activity perceived social support opportunities to have moderate direct impact on increasing PA outcomes, they advocated incorporating social networking features to foster a sense of community and accountability, and thus recommended as a priority “Do NOW”. The visual outputs from the prioritisation matrix activity are presented in supplementary Fig. 2.

### Stakeholder priorities for intervention design recommendations

#### Tailored educational modules on PA and menopause

##### Addressing PA literacy

Stakeholders identified a significant gap in PA literacy among middle-aged Saudi women, who often equate household chores with moderate-intensity PA, possibly due to the temporary fatigue they cause. However, these activities generally lack the sustained effort and muscle engagement necessary for health benefits.

*“We need to raise awareness about what counts as PA and what doesn’t. Some women reported exercising daily*,* but upon inquiry*,* they referred to household chores such as cleaning and cooking”* (Stakeholder 5, Family Medicine Doctor, WS2).

To address this, stakeholders recommended that the mHealth intervention deliver clear, accessible, and age-appropriate educational modules. These should explain structured PA and exercise emphasising intentional movement, sustained intensity, and progressive overload in adherence with PA guidelines, through engaging formats such as short videos, quizzes, and trackers.

*“The app should deliver bite-sized PA information via short videos and interactive materials*,* each with clear objectives and actionable steps*.*”* (Stakeholder 19, Fitness trainer, WS4).

##### Menopause-specific PA content

Stakeholders stressed the need for PA content to be meaningful and relatable that validates and addresses menopause-related challenges to help women feel understood and empowered.

*“I imagine the content of a PA app to be adapted… That means it would recognise menopausal symptoms in its content to help women feel more empowered.”* (Stakeholder 20, Fitness Trainer, WS4).

Stakeholders emphasised educating women on menopause-related symptoms such as fatigue, low energy, hot flushes, mood swings, and sleep disturbances, which could diminish motivation to engage in PA. Educational messaging should connect PA with symptom relief, including improved bone and muscle health, reducing pain, better mood, increased energy, and enhanced self-confidence.

*“We need to say it loud: PA helps women navigate menopause. We hear about its general benefits*,* but few act on it”* (Stakeholder 1, menopausal women, WS1).

##### Emphasising resistance training

Resistance training was seen as particularly beneficial but underutilised due to cultural misconceptions about its perceived safety for older women and appropriateness for women in general.

*“There is this idea that because we are older or just as a woman*,* resistance training or strength exercise might be inappropriate or even harmful. We need to get informed that it can help us feel better and live stronger”* (Stakeholder 3, menopausal women, WS1).

Stakeholders recommended promoting low-impact resistance exercises to address common age- and menopause-related concerns such as joint discomfort and body pain, and combat muscle loss (sarcopenia) and osteoporosis, using tools such as bodyweight, resistance bands, and modified positions to ensure accessibility and safety.

*“This phase [menopause transition] comes with some physical weakness due to estrogen drop*,* and naturally*,* as we age*,* muscles start to weaken gradually. Regular training helps reduce muscle decline”* (Stakeholder 20, Fitness trainer, WS4).

Stakeholders also highlighted resistance training in midlife as a cost-effective preventive measure that could reduce the need for later physiotherapy and enhance long-term health and resilience.

#### Adaptable yet structured exercise library

##### Home-based and culturally appropriate

Due to the high cost and limited access to women-only gyms in Saudi Arabia, stakeholders favoured home-based solutions.

*“We as clinicians can encourage women to join the gym*,* but high gym costs often make this inaccessible… Instead*,* doctors should consider recommending home-based activities where possible”* (Stakeholder 21, Policymaker and Practitioner, WS5).

HCPs also noted a lack of reliable, Arabic-language online PA resources available and specifically for women undergoing menopause or those with joint issues.

*“Not all online PA sources are safe or suitable or can be trusted to provide exercise programs for women in menopause*,* especially for women with knee or back pain”* (Stakeholder 9, Physiotherapist, WS3).

Consequently, stakeholders recommended an mHealth app with an adapted, evidence-based exercises tailored to midlife Saudi women, including low-impact and joint-friendly resistance exercise routines and progressive intensity options.

*“No fitness coaches address menopause or offer exercises for older groups… we need tailored programs for women in menopause”* (Stakeholder 20, Fitness Trainer, WS4).

*“From my experience*,* some women in this demographic have joint issues or limited mobility… modified positions should be offered*,* otherwise this might make things even worse*” (Stakeholder 18, Fitness Trainer, WS4).

Chair-based exercises were also proposed as alternatives for women with significant mobility constraints, though further evidence on their effectiveness is needed:

*“For women with knee pain or those who can’t walk due to issues like osteoarthritis*,* we could at least recommend chair-based exercises…”* (Stakeholder 6, Family Medicine Doctor, WS2).

##### Confidence-building and safety-focused tutorials

Fear of injury was identified as a major psychological barrier, described by one woman as an “invisible barrier (الحاجز الوه م) to PA” (Stakeholder 1, Menopausal Woman, WS1). This fear, rooted in prolonged sedentary lifestyles and limited exposure to structured PA, can intimidate midlife Saudi women, especially beginners.

*“The app should have a pre-beginner level bundle for those who’ve never exercised”* (Stakeholder 2, Menopausal Woman, WS1).

Stakeholders from fitness trainers recommended beginner-friendly guidance, warm-up and cool-down routines, and clear instruction on distinguishing “good” vs. “bad” pain. Features like slow-motion, audio-guided video demos, and repetition options were suggested to build user confidence and competence.

*“Many women have low PA skills. Instructions should be simple and repeated to build confidence”* (Stakeholder 20, Fitness Trainer, WS4).

#### Empathy-driven messaging

Stakeholders advocated for an empathetic approach in crafting PA messaging tailored to menopausal women, emphasising shifting away from traditional weight-loss centric messaging toward empathetic narratives focused on self-care, self-worth and emotional resilience during menopause.

*“It’s natural for a woman to see her routine change completely with the menopausal transition. Connecting PA with self-care*,* purpose*,* and self-worth can be more encouraging than focusing on aesthetics or weight loss”* (Stakeholder 12, Clinical Psychologist, WS3).

They advocated for using relatable stories of midlife Saudi women navigating menopause with PA and encouraging users to identify personal motivations.

*“Sharing authentic stories of local women engaging in exercise while navigating menopause can inspire others and help them find their motivation”* (Stakeholder 18, Fitness Trainer, WS4).

#### Progress-tracking and feedback mechanisms

Stakeholders stressed that the mHealth intervention features should prioritise celebrating personal progress with visual trackers, badges, and milestone recognitions rather than emphasising major physical transformations.

*“The app should build a narrative of hope and empowerment… focus away from body image to personal progress” (*Stakeholder 20, Fitness Trainer, WS4).

Incorporating compassionate prompts or affirmations reflecting improved mood, energy, and self-control was also recommended to emphasis the immediate psychological effects of exercise, reflecting a preference for menopause-relevant reinforcement that could help motivate sustained engagement.

*“Immediate benefits like feeling energised*,* or gaining self-control post-workout are often underrated but essential for motivation” (*Stakeholder 20, Fitness Trainer, WS4).

Consistently, stakeholders urged acknowledging menopausal symptoms compassionately, encouraging women to “*work with bodily changes rather than against them*” (Stakeholder 12, Clinical Psychologist, WS3). It appeared that none of the stakeholders proposed the inclusion of a feature for monitoring menopausal symptoms over time. This may suggest that symptom tracking was viewed as less essential within the proposed menopause-specific mHealth PA intervention.

#### Personalisation and customisation

Stakeholders reached a consensus that a one-size-fits-all approach is insufficient. Individual-level tailoring and flexible adjustments were seen essential to meet diverse menopausal women’s needs. Customisation and personalisation emerged as pivotal features in addressing the diverse requirements and conditions of midlife Saudi women.

*“Offer a range of exercises and adjustments to suit lifestyle and health conditions”* (Stakeholder 22, Policymaker, WS5).

Prioritised suggestions included onboarding questionnaires assessing joint pain, fatigue, PA levels, and preferences, setting individualised goals and plans.

*“It is important to offer personalised guidance… Initial assessment can create individualised plans based on specific cases and physical abilities”* (Stakeholder 20, Fitness trainer, WS4).

An agile, adaptive approach with flexible tools such as filtering exercise libraries and periodic reassessments was recommended to enhance relevance in long-term as circumstances evolve.

*“It would be useful to filter out the exercise library by condition like strengthening exercises after an injury*,* or exercises for weak knees or in case of arthritis. helps avoid overwhelm and boosts relevance”* (Stakeholder 1, menopausal women, WS1).

#### Social and community support features

Social and community components were discussed in most workshops. Many stakeholders noted the lack of social and emotional support hinders PA adherence for midlife Saudi women. Peer support groups, in-app chats, and moderated forums were encouraged to share common experiences and challenges, celebrate successes, and reduce isolation.

*“A community makes PA more enjoyable and keeps women connected”* (Stakeholder 19, Fitness Trainer, WS4).

Comparison and group-based PA challenges were viewed as desirable by some women: *“Imagine signing up for a monthly challenge with friends or even strangers you meet through the app… that would sound interesting”* (Stakeholder 2, menopausal woman, WS1). However, professional stakeholders expressed some reluctance, emphasising that such challenges must be carefully tailored to women’s abilities to avoid discouragement.

*“I would be worried if challenges are set to all users without adjusting to different women’s needs and physical abilities*,* as this might be discouraging or sometimes unsafe”* (Stakeholder 13, General Practitioner, WS2).

The idea of virtual exercise buddy matching gained traction, with stakeholders supporting the careful pairing of women with similar goals, interests, or proximity to provide mutual encouragement.

To respect cultural sensitivities, stakeholders recommended women-only forums, along with options for anonymous usernames to accommodate cultural privacy concerns. Notably, no significant concerns were raised about data security or information privacy during the workshops, which may suggest that social and cultural norms surrounding privacy were more influential in this context.

#### Culturally tailored platform

Stakeholders recognised increasing acceptance of PA among Saudi women but noted older generations face entrenched cultural norms emphasising modesty and perceptions of women as delicate, limiting exercise engagement.

*“Women in their fifties and sixties grew up where exercise was discouraged and seen as inappropriate as societal norms emphasised modesty and portrayed women as delicate*,* or soft”* (Stakeholder 22, Policymaker, WS5).

They suggested adapting the intervention to fit the Saudi cultural context to support socially acceptable, long-term PA adherence. For example, dance, a culturally familiar and enjoyable activity, was suggested as a possible gateway to PA participation for midlife Saudi women.

*“Many middle-aged women enjoy dancing*,* like belly dance classes*,* which could encourage PA”* (Stakeholder 7, Family medicine, WS2).

Stakeholders emphasised aligning PA messaging with Islamic principles and religious practices. Suggestions included incorporating Quranic verses that promote health and framing PA as essential for maintaining bone, muscle, and joint health to support comfortable worship (daily prayer ‘*Salah’*). This idea resonated strongly with menopausal women in Workshop 1, where one participant shared how strength and flexibility helped her perform bowing (*Rukoo’*) and prostration (*Sujood*) more comfortably. This sparked broader discussion on how PA, particularly strength and flexibility training, could enhance spiritual fulfilment and motivation by connecting exercise to deeply held cultural and religious values.

Stakeholders also emphasised the need for the intervention to use simple Arabic language, local dialects, and familiar analogies to describe exercises. For example, “*pull up as if putting something on a high shelf” or “push forward as if closing a heavy door*” HCP, WS2, to simplify exercise forms. Fitness trainers recommended using culturally appropriate exercise references, such as rebranding yoga as “stretching and flexibility exercises” due to local associations of yoga with non-Islamic ritual practices rather than fitness purposes.’ due to its local association with ritual rather than fitness. Stakeholders also advised using modest visuals and non-revealing attire of trainers to align with conservative cultural and religious norms, thereby fostering acceptance and supporting long-term engagement.

### Integration of the optimised menopause-specific PA app in Saudi Arabia: implementation supporting strategies

While the design recommendations discussed above define what the intervention should include, stakeholders in the final workshop shifted their focus toward how the mHealth intervention could be effectively integrated and sustained within existing health and social systems in Saudi Arabia. They concurred that promoting PA among menopausal Saudi women requires a holistic ecosystem approach that embeds the mHealth app within users’ lived realities and aligns it with broader structural systems to support sustained engagement and intervention sustainability.

*“A holistic approach can ensure Saudi women receive the full range of support they need and not promote PA in isolation of their local context”* (Stakeholder 22, Policymaker).

Stakeholders reported that while most midlife Saudi women today are educated and familiar with smartphones, digital literacy varies and might pose challenges for some users, highlighting the need for simplified app design with intuitive navigation and step-by-step tutorials. A blended delivery model was also proposed, combining in-person induction with ongoing virtual support, and offering in-app help desks, training, and community-based young female “tech ambassadors”. However, due to social privacy concerns, the representative woman expressed a preference for family-based assistance (e.g., from a daughter or granddaughter).

*“…Today’s generation of women in their fifties are mostly educated*,* many of us have jobs and are more likely to use smartphones for everyday activities and look up answers online*,* whether it’s about health*,* exercise*,* or something else*” (Stakeholder 1, menopausal women).

*“Some may need more guidance in person or at least on a video tutorial” (Stakeholder 20*,* Fitness trainer)*.

Involving trusted institutions, particularly the Saudi MOH and multidisciplinary HCPs, was highlighted as crucial for credibility and trust. Co-creation of intervention content with Saudi-based experts and promoting the mHealth app via primary care settings was emphasised as an optimal entry point for menopausal women seeking health advice. One GP proposed quick-reference materials to streamline app promotion during clinical visits: “*Menopause can be overlooked […]. It could save time for both doctors and patients*,* but we need a manual or a quick guide for doctors to promote the app usage through screening a QR code or distributing pamphlets*” (Stakeholder 10, GP).

MOH oversight was identified as a beneficial long-term strategy to enhance public trust and remove financial barriers. However, the group discussion revealed that government endorsement may require prolonged administrative processes. Integration with the national health platform (Sehhaty “MyHealth”) was also viewed positively, providing opportunities for personalised care and adherence to the intervention: *“If my doctor knows I’ve made progress on my PA over the last three months*,* possibly by integrating my data with the Sehhaty app*,* it could make routine consultations with my GP more productive*” (Stakeholder 1, menopausal woman). Yet, stakeholders from a policymaker and an app designer cautioned that data privacy regulations and technical interoperability would present challenges requiring substantial policy alignment.

Cross-sector partnerships among public health, sport authorities, fitness centers, faith-based and non-profit organisations were viewed essential for dissemination. Large media campaigns were recommended but may be resource-intensive; collaborating with popular social media influencers targeting middle-aged Saudi women was suggested as an effective alternative for raising awareness and intervention uptake: “*Awareness should begin on social media*,* but health and fitness content is rarely featured by the popular figures this demographic follows*,* who mostly focus on fashion and food*” (Stakeholder 20, fitness trainer).

During feasibility assessment, stakeholders reflected on the practicality of the proposed strategies for supporting intervention implementation. Intuitive interface design, onboarding tutorials, family-based digital support, HCPs involvement in content development, and social media campaigns were identified as high-impact immediate goals likely to enhance digital literacy and acceptability for taking up the intervention. Blended delivery approaches and promotion via primary care pathways and national mass media campaigns were categorised as medium-term goals that could perhaps improve intervention accessibility and engagement. Integration with the national health platform and the establishment of sustained public-private partnerships were recognised as longer-term objectives, requiring further regulatory alignment and direct governmental support.

**Fig. 4:**
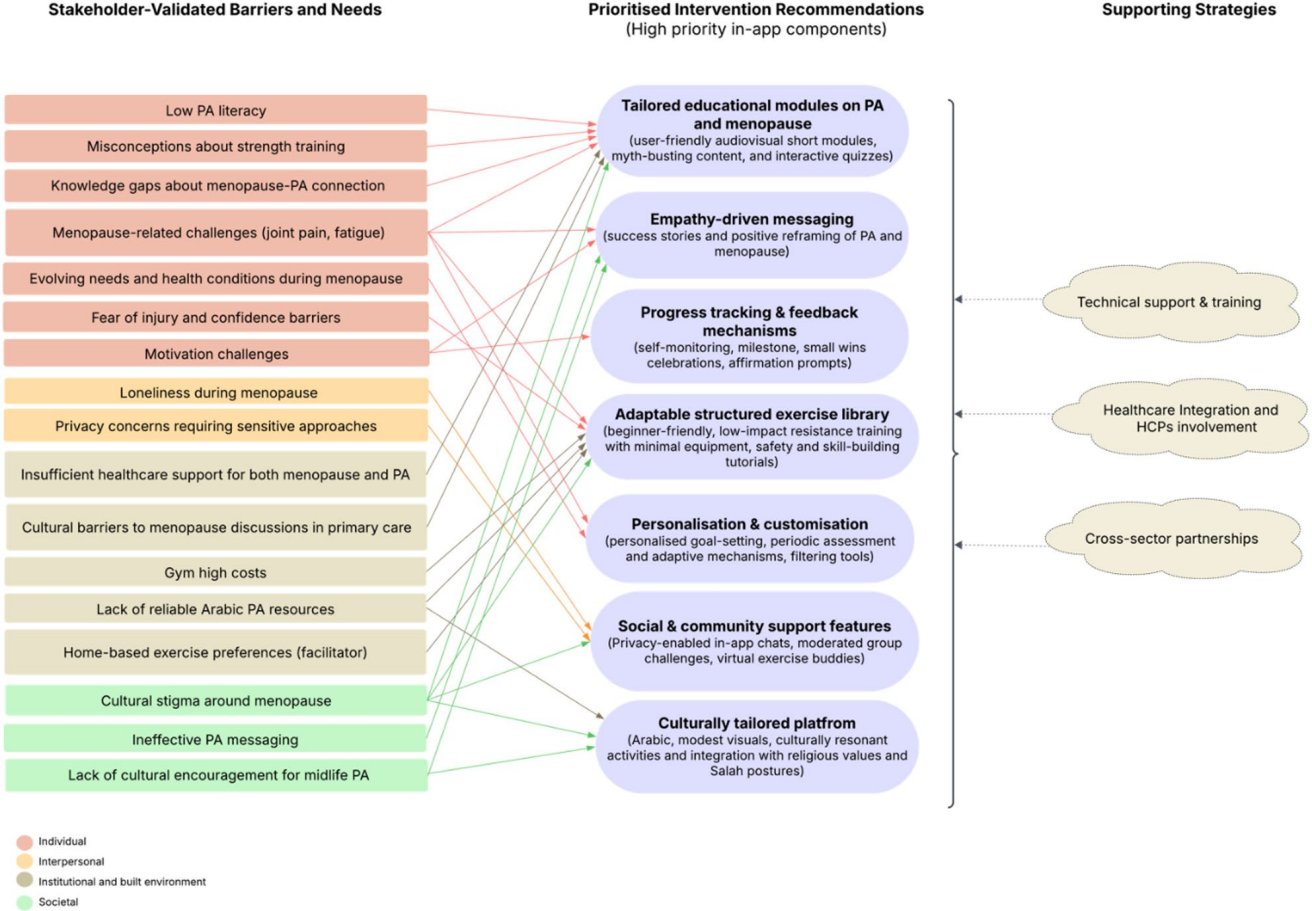
A co-designed conceptual framework guiding optimisation and integration of a menopause-specific PA mHealth-based intervention in Saudi Arabia

Together, the prioritised intervention design recommendations and implementation strategies identified through the stakeholder discussions informed the development of a co-designed conceptual framework for contextually tailoring an mHealth PA intervention for midlife Saudi women experiencing menopause (Fig. 4). While grounded in stakeholder input, the framework was derived from rather than co-created during the co-design workshops. It serves as a practical guide for optimising and integrating a culturally tailored mHealth intervention to support PA among menopausal Saudi women.

### COM-B MAPPING

The behavioural mapping reveals clear patterns in how the stakeholder co-designed recommendations aligned with different COM-B components [36]. The analysis suggests theoretical coherence in stakeholder priorities and highlights areas where the co-designed recommendations may be less represented. All six COM-B components (physical and psychological capability, reflective and automatic motivation, and physical and social opportunity) appear to be addressed through the co-designed, prioritised intervention design recommendations in a synergistic way, although the level of emphasis varies between primary and indirect pathways. Primary pathways refer to the most direct mechanisms through which each co-designed recommendation is expected to influence behaviour, while indirect pathways refer to additional supporting processes that may occur alongside the primary mechanisms creating complementary effects. Detailed tabulations are provided in supplementary file 3.

The mapping suggests that the co-design process with stakeholders helped translate lived experiences into concrete recommendations that align with established behaviour change theory [36]. The range of TDF domains reflected in the recommendations, including knowledge, skills, beliefs about consequences, identity, reinforcement, and emotion, implies that stakeholders recognised behaviour change as multifaceted and produced recommendations spanning multiple COM-B pathways. However, there appears to be a concentration of recommendations targeting psychological capability and reflective motivation. This pattern aligns with the priorities voiced during co-design but may need to be complemented with additional strategies that support long-term behaviour change, such as attention to habit formation strategies and broader environmental restructuring.

The intervention functions within the BCW appear to be engaged predominantly through education, enablement, persuasion, and training. These functions are evident across the co-designed intervention design recommendations and supporting strategies, reflecting stakeholder emphasis on knowledge provision, skill development, and motivational framing. Approaches such as coercion, restriction, and legislation do not appear in the co-designed recommendations, which is consistent with the participatory nature of the process and its focus on voluntary PA behaviour change. The supporting strategies introduce policy categories that extend beyond individual-level intervention, suggesting that stakeholders recognised that sustainable PA behaviour change requires structural enablers beyond the digital intervention. Technical support and training to address digital literacy barriers, healthcare integration to enhance professional credibility and routine embedding, and cross-sector partnerships were proposed as potential mechanisms to support shifts in social norm through established and trusted community structures.

Mapping the recommendations onto COM-B, TDF, and BCW provided a structured lens to evaluate the comprehensiveness of the stakeholder co-designed recommendations in potentially addressing capability, motivation, and opportunity components relevant to influencing PA behaviour. The mapping was also useful to highlight areas where the recommendations could be strengthened and indicated that linking them to behaviour change frameworks can serve as a basis for iterative logic model refinement and enhancing transparency and replicability. Further refinement should be guided by continued stakeholder engagement and evidence from real-world implementation.

## Discussion

### Principal findings

This paper reports the co-design process aimed at developing an mHealth-based intervention to support PA among midlife Saudi women experiencing menopause. Multidisciplinary stakeholders collaboratively identified key intervention recommendations and implementation supporting strategies, outlining how tailoring can be applied when designing mHealth PA interventions for this population. The co-designed recommendations emphasised the importance of tailoring for menopause-related needs and life-stage considerations, cultural sensitivity and alignment, and varied digital literacy and skills. The co-designed recommendations are consistent with the COM-B model, which posits that increasing capability, motivation, and opportunity supports behaviour change [36]. These participatory insights may help inform the future development and optimisation of acceptable and potentially engaging mHealth PA interventions for midlife women in Saudi Arabia.

### Comparison with previous work

A recent review on PA during the menopausal transition emphasises the need for tailored strategies that promote empowerment, highlight menopause-specific health benefits, support self-efficacy and injury prevention, facilitate social support, and ensure equitable access to suitable opportunities [47]. These priorities correspond with the recommendations generated in the present workshops, including menopause-specific educational modules, empathetic messaging, adaptable exercise libraries, social interaction options, and measures to support digital skills and inclusivity in mHealth tools. Similar considerations were identified in the Australian Active Women over 50 co-design work, which stressed the importance of life-stage relevant information, realistic goals, accountability, and non-guilt framing [48, 49], suggesting that midlife women across contexts encounter comparable capability and motivation-related barriers. Consistent with evidence from Ireland positioning PA as self-care and mental wellbeing support [50], stakeholders in this study recommended framing PA around self-care and resilience rather than appearance or weight loss focused messaging. These perspectives align with women-centred approaches that recognise lived experiences and biopsychosocial influences during menopause [51, 52]. Concerns related to ageing, body image, and stigma, which emerged in our earlier qualitative work with Saudi midlife women [33], may constrain perceived capability, increase self-blame, and reinforce disengagement from PA.

Supporting the focus on user-centred design, recent qualitative analysis of user-generated posts on the X platform related to five popular fitness apps identified negative emotional responses, including shame, guilt, and demotivation, suggesting that such apps may produce unintended emotional or behavioural consequences and potentially contribute to disengagement [53]. Although based in Western contexts, these findings align with insights from the current co-design workshops, where stakeholders generated and prioritised strategies such as empathetic messaging, menopause-relevant content, flexible goal setting, adaptable activity plans, and cultural appropriateness. Collectively, these perspectives could suggest that reliance on numerical tracking and rigid goal-setting features, which are common in many fitness apps, may be viewed less desirable and less suitable for supporting sustained PA participation and wellbeing among midlife women.

Promoting resistance training emerged as a particularly valued component, reflecting evidence underscoring its importance for midlife and ageing women, those at risk of sarcopenia or osteoporosis [54, 55]. Stakeholder suggestions for graded progression, safety cues, and self-paced controls mirror recommendations from a review calling for strength training interventions for women that address misconceptions, emphasise personally meaningful benefits, and adapt activities to available resources [56]. Comparable participatory research with people living with chronic health conditions has highlighted the importance of flexible intensity, duration, and delivery to support adherence [57]. The workshops insights also align with international evidence showing that flexibility and individual-level tailoring are key to improving PA uptake among older adults [58, 59]. Personalisation and adaptive features were seen as necessary to accommodate differences in menopause trajectories, health conditions, baseline fitness levels, and prior exercise experiences, supporting autonomy as circumstances evolve [60].

Stakeholders welcomed social support options such as moderated forums and buddy systems, though interest in comparison or competition was mixed, reflecting concerns that variations in symptoms and physical capacity could make comparison discouraging. The preference for self-comparison aligns with findings from adults and older adults using PA apps [60–62]. A previous mHealth PA intervention study targeting midlife American women have reported low engagement with community forums, highlighting the need for active facilitation to sustain participation [63, 64]. Similarly, other co-design research has recommended clear participation criteria and the involvement of moderators and facilitators drawn from local midlife women’s communities [48, 49]. These insights suggest that effective online peer support depends on ongoing facilitation and community building rather than platform design alone.

This study contributes to limited evidence on how cultural tailoring can be operationalised for mHealth PA interventions targeting Saudi midlife women. Although cultural sensitivity is widely emphasised, detailed reporting on adaptation processes is often lacking [65]. Our workshops indicated that cultural tailoring extends beyond language translation or modest imagery, requiring strategies addressing cultural beliefs and norms that influence both menopause and PA behaviours. Suggested approaches included reframing menopause terminology using positive Arabic expressions, integrating faith-aligned motivational cues, offering modest and home-based exercises, and enabling anonymous participation. These strategies reflect structural tailoring as described by Resnicow et al. [66]. Similar culturally tailored design principles have been employed in PA interventions for African American midlife women, which used culturally familiar role models and identity-reflective voiceovers [63], and for Arab women in Australia, where culturally meaningful content and women-only delivery supported acceptability [67]. Stakeholders in our study extended these insights by proposing the integration of Islamic self-care values, such as linking resistance training benefits to comfort during prayer movements.

Stakeholders also emphasised the importance of embedding the mHealth intervention within broader health and social system pathways rather than treating it as a standalone digital tool. Proposed strategies included encouraging family-mediated digital assistance, supporting mHealth referral pathways within primary care, integrating with the MOH’s national mHealth platform, and establishing cross-sectoral partnerships. These recommendations for implementation correspond with recent qualitative work with policymakers in Saudi Arabia, which identified mHealth technologies as promising for PA promotion but highlighted the need for coordinated government oversight, technical capacity, and collaboration across sectors [68]. Such insights reflect a socio-technical perspective in which mHealth interventions operate within systems shaped by cultural norms, infrastructure, and governance [69, 70]. Family-mediated digital support appeared more acceptable than external community champions, contrasting with Western models that employ peer navigators or lay health workers [71, 72]. This suggests that participatory co-design can help identify culturally grounded strategies for digital inclusion that broader frameworks may overlook.

The workshops also highlighted potential risks. Integrating the intervention into healthcare pathways may unintentionally contribute to the medicalisation of menopause, shifting PA from a self-care practice to a treatment narrative. While professional endorsement may normalise help-seeking, careful implementation is required.

### Co-design process limitations and strengths

This study represents one of the first applications of a structured co-design approach aimed at digital health intervention development and optimisation in the Saudi Arabian context. However, several limitations should be acknowledged and warrant critical reflection. First, a key limitation concerns the limited number of midlife Saudi women who participated in the group-based workshops; notably, three engaged actively and only one woman acted as a representative and took part in the cross-sectoral prioritisation workshop, despite multiple invitations being extended to eligible participants from the earlier interview phase. This limited end-user representation in the co-design activities may have influenced the power dynamics and reduced the diversity of women’s voices in shaping or prioritising recommendations, potentially skewed towards professional perspectives emphasising feasibility and implementation practicality. However, this limitation was partially mitigated by the preceding phase of 29 in-depth, one-one-one interviews and think-aloud sessions, through which women’s perspectives, lived experiences and mHealth expectations had already been captured and used to inform workshop materials and recommendation direction.

It is also possible that cultural norms around modesty and social privacy made some women less comfortable to engage in group-based settings, a consideration raised by our PPI members, which may have contributed to the limited recruitment and representation of women in the workshops. This points to a potential tension between participatory co-design aspirations and the contextual sociocultural barriers to group-based participation among Saudi midlife women. While broader end-user participation would have strengthened the prioritisation stage, it is worth noting that Workshop 5 was primarily intended to refine rather than generate new recommendations. Nevertheless, it remains critically important that future development phases validate the final prioritised recommendations with larger and more diverse samples of menopausal Saudi women.

Second, the exclusive use of online workshops, although pragmatic and logistically advantageous, the virtual format may have reduced opportunities for spontaneous interaction, informal rapport-building, and creative exchange. It may also have shaped participation dynamics by favouring individuals with higher digital confidence, potentially influencing whose voices were most prominently expressed. These considerations highlight digital literacy and accessibility as concerns relevant to both research methodology and intervention design, suggesting that future co-design and implementation phases should incorporate hybrid engagement strategies and more targeted digital inclusion approaches.

Third, the absence of direct engagement with key community actors, such as social and religious leaders, may have limited opportunities for deeper cultural validation and broader community endorsement. Nevertheless, local stakeholders likely drew upon both their personal and professional perspectives throughout the co-design process, partially reflecting broader community values.

Finally, although informal feedback from participants was positive and some professional stakeholders requested copies of the storyboards used during the workshops, the co-design process was not formally or anonymously evaluated. This may have limited the extent to which stakeholders felt comfortable sharing critical feedback. This reflects the study’s primary focus on generating tailored intervention design recommendations rather than systematically assessing the co-design process itself within cultural contexts. Embedded reflexive practices, including facilitator observations, documentations, and iterative adaptations of workshop topic guide, helped support process transparency. However, these approaches do not substitute for formal evaluation of inclusivity, participant empowerment, and satisfaction. Future participatory research should therefore incorporate more systemic process evaluation to inform best practices in co-design for digital health interventions in conservative and underrepresented contexts.

Co-design methodologies remain relatively underexplored in Saudi Arabia and the wider MENA region, and this study represents a meaningful contribution to participatory health innovation in this context [73]. A key strength was the inclusion of a diverse range of stakeholders, including HCPs, fitness trainers, an app developer, policymakers, and menopausal women themselves, ensuring that intervention recommendations were informed by both lived experience and professional expertise. The use of homogenous stakeholder groups in the early co-ideation workshops helped reduce power imbalances and fostered inclusive, safe spaces where all voices could be heard [31].

Additionally, the invitation of a midlife Saudi women’s representative to participate in professional sessions helped maintain a connection to real-life intervention needs and enabled a meaningful exchange of perspectives. However, her contribution was not possible in Workshop 2 due to a language barrier, as that session was conducted in English to accommodate non-Arabic-speaking professionals. We carefully monitored group interactions to ensure that women’s voices remained central to the process. Co-facilitation further supported active participation across stakeholder groups and contributed to more equitable involvement in idea generation and decision-making, which are core principles of co-design [74, 75]. The sequential approach and the integration of our earlier qualitative interview findings with midlife Saudi women into visual storyboards helped enrich workshop discussions and made abstract ideas more accessible [76].

Conducting workshops online appeared to facilitate participation across geographic and professional boundaries, overcoming logistical challenges and enabling flexible language use among diverse groups. Similar applications of online co-design have been documented in the literature, particularly within digital health projects [77].

The final workshop incorporated a structured prioritisation activity that supported active engagement and transparent decision-making. The 2 × 2 matrix is a commonly used tool in design thinking [78] and has been applied for stakeholder mapping across a range of settings [79]. The application of APEASE criteria during the prioritisation workshop further supported a structured and transparent approach to decision making [36]. Additionally, mapping co-design outputs onto COM-B, TDF, BCW intervention functions enhanced the theoretical robustness and replicability of the proposed intervention model [36].

### Reflections on the co-design outcomes and implications

Our critical reflection on the co-design workshops, informed by the subsequent theoretical mapping, identified areas requiring more explicit attention in future work. First, although stakeholders recognised barriers to PA during menopause across individual, social, and environmental levels, many of the co-designed and prioritised ideas focused on addressing women’s knowledge, confidence, and motivation. This pattern was further reflected in the behavioural mapping, which indicated a concentration on psychological capability and reflective motivation. This concentration may have been unintentionally influenced by several factors. For example, the focus on mHealth as the primary intervention modality, alongside the feasibility-oriented prioritisation approach adopted in the final workshop, may have favoured more immediate and implementable individual-level strategies. Alternatively, it may suggest that stakeholders implicitly assumed that PA behaviour change could be achieved primarily through individual-level approaches, or that they prioritised short-term barriers to initiating PA over the less immediately experienced challenges of sustaining behaviour over time.

Addressing women’s knowledge and self-efficacy in relation to exercise and menopause is undoubtedly important; yet evidence indicates that information provision alone rarely drives behaviour change unless embedded within broader supportive environments [36]. Among menopausal women in particular, environmental restructuring strategies are considered essential for supporting PA behaviour change [47]. Future co-design activities should therefore more explicitly scaffold ecological and structural level strategies alongside individual-level ones. Notably, participating stakeholders did acknowledge systemic and institutional-level factors, including healthcare integration and cross-sectoral partnerships, as essential enabling strategies for the menopause-specific PA mHealth intervention uptake and engagement.

Second, family-based support, identified in the preceding qualitative interview study as a key facilitator of PA engagement among midlife Saudi women. Particularly, spousal and daughter support were notably absent from stakeholder discussions during the co-design workshops, apart from family digital support. This absence may reflect sensitivities around discussing family matters in group settings, or around the specific context of menopause-focused intervention, which may be viewed as less appropriate to share with family members [33]. Given that family dynamics represent a powerful enabler of PA participation among women in collectivist cultures such as Saudi Arabia [80], family-based exercise features or family support mechanisms may represent a missed social opportunity warranting further attention in subsequent co-design research.

Additionally, data privacy and security concerns received limited attention during the workshops, despite being a well-established issue in mHealth research [81]. This may reflect stakeholders’ limited technical backgrounds, or conversely, high confidence in national data protection regulations and MOH oversight, an assumption that appeared to be reinforced during workshop discussions of potential regulatory supervision. Similar patterns have been observed in co-design studies with older adults [82], suggesting that data privacy and trust may require more deliberate exploration and prompting in future co-design work, and would benefit from the involvement of stakeholders with data regulation or legal expertise.

### Next steps and future research directions

In line with the updated Medical Research Council (MRC) framework [83], the present co-design study represents a pre-development stage in the intervention development process. The immediate next step - developmental phase - will involve further iterative refinement of the co-designed recommendations, informed by the COM-B and BCW mapping analysis undertaken, to strengthen the model’s anticipated behavioural mechanisms, addressing the under-emphasised mechanisms of habit formation and broader environmental restructuring, and translate these recommendations into specific, tangible intervention design features. Subsequent iterative co-design and PPI activities are expected to engage a broader range of end-users and community stakeholders in the co-production of specific intervention content, specify active ingredients using the Behaviour Change Technique Ontology [84], and support functional prototyping.

As part of formative evaluation and empirical testing, a mixed-methods process evaluation should be incorporated to assess the feasibility and acceptability of the co-designed, tailored intervention with the intended users, and to collect preliminary outcome data. Such data could draw on Ecological Momentary Assessment (EMA), a self-report method for capturing real-time or near real-time data on women’s PA, mood, and symptoms in everyday settings. This stage should also explore user experiences and engagement patterns, including minimum meaningful engagement thresholds, user satisfaction, and preliminary effects on targeted outcomes, as well as the fidelity of key intervention features, such as menopause-specific PA education, personalisation, social support, and cultural tailoring. It should further examine which hypothesised behavioural mechanisms (e.g., enhancing psychological capability, reflective motivation, and social opportunity) were supported and which were not. Process evaluation findings should then be mapped against the logic model to inform further refinement of intervention content and messaging prior to larger-scale effectiveness trials.

Aligning with the user-centred and theoretically integrated approach adopted in this research, the Person-Based Approach (PBA) provides a systemic framework for maintaining a log of changes throughout the intervention optimisation, evaluation, and implementation stages [85]. The PBA table of changes can be used to document modifications arising from ongoing stakeholder and PPI engagement and from qualitative process evaluation findings to ensure that the intervention remains contextually relevant and centred on the needs and preferences of midlife Saudi women.

In summative evaluation trials, the co-designed, menopause-specific PA mHealth intervention should be evaluated for its long-term effects beyond short-term and preliminary benefits. Key outcomes should include PA adherence and habit formation, changes in menopause-related symptoms, and quality-of-life outcomes. Generating such evidence will be essential for establishing the intervention’s effectiveness among midlife Saudi women and for supporting wider adoption.

While the current co-designed recommendations identified system-level contextual factors as supporting strategies for implementation, further implementation planning with key stakeholders remains necessary. Subsequent real-world implementation research should examine practical challenges related to embedding the envisioned mHealth intervention within existing primary care services, national public health initiatives, and community pathways, to inform future scalability. This may include conducting primary research with primary care practitioners and other relevant stakeholders regarding intervention use, and testing the potential influence of organisational, policy, regulatory, and technical factors on the menopause-PA intervention’s reach, uptake, and sustainability. The integration of established implementation frameworks, such as the Normalisation Process Theory (NPT), would usefully inform and guide the examination of implementation strategies to be adopted [86].

The culturally tailored design recommendations generated may guide the development of other digital public health solutions in Saudi Arabia, supporting broader health transformation goals aligned with Saudi Vision 2030 [87, 88]. Furthermore, our findings may be transferable to other comparable cultural contexts from Arab and Muslim-majority communities, providing a foundation for the development of menopause-focused PA and health promoting mHealth tools, subject to further contextual adaptation.

## Conclusions

This study demonstrates the utility of co-design workshops as an acceptable and feasible approach for generating concrete recommendations to design culturally appropriate and potentially motivating mHealth solutions to support PA among midlife Saudi women, an underrepresented population and setting.

Through an iterative collaborative process, contextually tailored, high-level intervention components and system-level implementation strategies were generated in relation to the broader social and health sysem context. These design recommendations emphasised on tailoring the mHealth PA intervention to the menopause life stage, the Saudi cultural context, and the digital literacy needs of midlife Saudi women. A preliminary, user-centred, theory-informed conceptual framework was developed through synthesis of the co-design findings, serving as a blueprint for future intervention development. The empirically grounded recommendations are intended to work synergistically to support behaviour change, offering actionable guidance for developers, clinicians, and policymakers seeking to design more inclusive digital health solutions for midlife women. However, the potential effectiveness of the recommended intervention features in supporting sustained PA behaviour change remains uncertain and requires empirical testing.

Future research should test whether these culturally grounded, evidence-informed recommendations can be translated into effective app features that support sustained PA behaviour change and improved health outcomes. The challenge ahead lies in moving from exploration to design and delivery, ensuring that mHealth innovation reaches the women most in need of support during this critical life transition, not only those with existing access and literacy, thereby helping to reduce social inequalities. If successfully implemented, this approach may also offer transferable lessons for other cultural contexts where similar gaps exist.

## Supplementary Information

Supplementary Material 1.

Supplementary Material 2.

Supplementary Material 3.

## Data Availability

The datasets generated the qualitative co-design study (consisting of group workshop transcripts) are not publicly available due to the sensitive nature of the data and confidentiality commitments to participants. However, the deidentified data are available for research purposes from the corresponding author on reasonable request.

## Abbreviations

APEASE: Affordability, Practicability, Effectiveness/cost-effectiveness, Acceptability, Side effects/safety, and Equity
AI: Artificial intelligence
BCTs: Behaviour Change Techniques
BCW: Behaviour Change Wheel
COM-B: Capability, Opportunity, Motivation - Behaviour
EMA: Ecological Momentary Assessment
HCP(s): Healthcare providers
MENA: Middle East and North Africa
MOH: Ministry of Health
MRC: UK Medical Research Council
NCDs: Non-communicable diseases
PA: Physical activity
PBA: Person-Based Approach
PPI: Public and Patient Involvement
QCA: Qualitative Content Analysis
TDF: Theoretical Domains Framework
WHO: World Health Organization

## References

[1] World Health Organization. 2018. *Global action plan on physical activity 2018–2030: More active people for a healthier world*. Geneva: World Health Organization. https://www.who.int/publications/i/item/9789241514187. Accessed at 13 April 2025.

[2] Kim MJ, Cho J, Ahn Y, Yim G, Park HY. Association between physical activity and menopausal symptoms in perimenopausal women. BMC Womens Health. 2014;14:122. 10.1186/1472-6874-14-122 . PMID: 25277534; PMCID: PMC4287540. 25277534 10.1186/1472-6874-14-122 PMC4287540

[3] Khan KM, Thompson AM, Blair SN, Sallis JF, Powell KE, Bull FC, Bauman AE. Sport and exercise as contributors to the health of nations. Lancet. 2012;380(9836):59–64. 10.1016/S0140-6736(12)60865-4. PMID: 22770457. 10.1016/S0140-6736(12)60865-4 22770457

[4] Sternfeld B, Dugan S. Physical activity and health during the menopausal transition. Obstet Gynecol Clin North Am. 2011;38(3):537–66. 10.1016/j.ogc.2011.05.008 . PMID: 21961719; PMCID: PMC3270074. 21961719 10.1016/j.ogc.2011.05.008 PMC3270074

[5] Money A, MacKenzie A, Norman G, et al. The impact of physical activity and exercise interventions on symptoms for women experiencing menopause: overview of reviews. BMC Womens Health. 2024;24:399. 10.1186/s12905-024-03243-4. 39003439 10.1186/s12905-024-03243-4 PMC11245773

[6] Wasley D, Gailey S. Menopause and the role of physical activity - The views and knowledge of women aged 40–65. Post Reprod Health. 2024;30(2):77–84. doi: 10.1177/20533691241235273. Epub 2024 Feb 23. PMID: 38393976; PMCID: PMC11188563. 10.1177/20533691241235273 PMC11188563 38393976

[7] Sport England. Supporting women to get active during menopause. 2017. [Research Report]. Available at: https://womeninsport.org/wp-content/uploads/2018/05/Menopause-summary-report-FINAL.pdf. Accessed at 13 April 2025.

[8] Sport England. Women in Sport. Menopause, Me, and Physical Activity 2018. (pp. 1–20) [Research Report]. Available at: https://womeninsport.org/wp-content/uploads/2018/05/Menopause-report-PDF-final-1-2.pdf. Accessed at 13 April 2025.

[9] Im E-O, Chee W, Lim H-J, Liu Y, Kim HK. Midlife Women’s Attitudes Toward Physical Activity. J Obstetric Gynecologic Neonatal Nurs. 2008;37(2):203–13. 10.1111/j.1552-6909.2008.00219.x. 10.1111/j.1552-6909.2008.00219.x 18336444

[10] Kelly S, Martin S, Kuhn I, Cowan A, Brayne C, Lafortune L. Barriers and Facilitators to the Uptake and Maintenance of Healthy Behaviours by People at Mid-Life: A Rapid Systematic Review. PLoS ONE. 2016;11(1):e0145074. 10.1371/journal.pone.0145074. 26815199 10.1371/journal.pone.0145074 PMC4731386

[11] McArthur D, Dumas A, Woodend K, Beach S, Stacey D. Factors influencing adherence to regular exercise in middle-aged women: A qualitative study to inform clinical practice. BMC Women’s Health. 2014;14(1). 10.1186/1472-6874-14-49. 10.1186/1472-6874-14-49 PMC3975263 24666887

[12] Tinker A, Molloy L, Monks I, Pennells L, Russell E, Haines E, THE BENEFITS AND BARRIERS OF EXERCISE FOR THE PHYSICAL HEALTH OF OLDER WOMEN. J Aging Res Lifestyle. 2017;1–7. 10.14283/jarcp.2017.6.

[13] Yarwood J, Carryer J, Gagan MJ. Women maintaining physical activity at midlife: Contextual complexities. Nurs Prax N Z Inc. 2005;21(3):24–37. 16764159

[14] Al-Hazzaa HM. Physical inactivity in Saudi Arabia revisited: A systematic review of inactivity prevalence and perceived barriers to active living. Int J Health Sci (Qassim). 2018 Nov-Dec;12(6):50–64. PMID: 30534044; PMCID: PMC6257875. PMC6257875 30534044

[15] Al-Eisa ES, Al-Sobayel HI. Physical Activity and Health Beliefs among Saudi Women. J Nutr Metab. 2012;2012:642187. doi: 10.1155/2012/642187. Epub 2012 Feb 22. PMID: 22523673; PMCID: PMC3317126. 10.1155/2012/642187 PMC3317126 22523673

[16] Abdelhay O, Altamimi M, Abdelhay Q, Manajrah M, Tourkmani AM, Altamimi M, Altamimi T. Perceived barriers to physical activity and their predictors among adults in the Central Region in Saudi Arabia: Gender differences and cultural aspects. PLoS One. 2025;20(2):e0318798. doi: 10.1371/journal.pone.0318798. Erratum in: PLoS One. 2025;20(9):e0331572. 10.1371/journal.pone.0331572. PMID: 39919050; PMCID: PMC11805373. 10.1371/journal.pone.0318798 PMC11805373 39919050

[17] General Authority for Statistics. Saudi Arabia. Physical Activity Statistics Saudi Publication. 2024. https://www.stats.gov.sa/en/statistics-tabs?tab=436312&category=1278616. Accessed at 12 Novemeber 2025.

[18] General Authority for Statistics. Saudi Arabia: Communications and information technology report. Riyadh: GASTAT. 2023. https://www.stats.gov.sa/en/. Accessed at 12 April 2025.

[19] Romeo A, Edney S, Plotnikoff R, Curtis R, Ryan J, Sanders I, Crozier A, Maher C. Can Smartphone Apps Increase Physical Activity? Systematic Review and Meta-Analysis. J Med Internet Res. 2019;21(3):e12053. 10.2196/12053 . PMID: 30888321; PMCID: PMC6444212. 30888321 10.2196/12053 PMC6444212

[20] Feter N, Dos Santos TS, Caputo EL, da Silva MC. What is the role of smartphones on physical activity promotion? A systematic review and meta-analysis. Int J Public Health. 2019;64(5):679–90. 10.1007/s00038-019-01210-7 . Epub 2019 Feb 13. PMID: 30758514. 30758514 10.1007/s00038-019-01210-7

[21] Alley SJ, Waters KM, Parker F, Peiris DLIHK, Fien S, Rebar AL, Vandelanotte C. The effectiveness of digital physical activity interventions in older adults: a systematic umbrella review and meta-meta-analysis. Int J Behav Nutr Phys Act. 2024;21(1):144. 10.1186/s12966-024-01694-4 . PMID: 39696583; PMCID: PMC11658456. 39696583 10.1186/s12966-024-01694-4 PMC11658456

[22] AlSwayied G, Guo H, Rookes T, Frost R, Hamilton FL. Assessing the Acceptability and Effectiveness of Mobile-Based Physical Activity Interventions for Midlife Women During Menopause: Systematic Review of the Literature. JMIR Mhealth Uhealth. 2022;10(12):e40271. 10.2196/40271 . PMID: 36485026; PMCID: PMC9789501. 36485026 10.2196/40271 PMC9789501

[23] Naderbagi A, Loblay V, Zahed IUM, Ekambareshwar M, Poulsen A, Song YJC, Ospina-Pinillos L, Krausz M, Mamdouh Kamel M, Hickie IB, LaMonica HM. Cultural and Contextual Adaptation of Digital Health Interventions: Narrative Review. J Med Internet Res. 2024;26:e55130. 10.2196/55130. 38980719 10.2196/55130 PMC11267096

[24] Malloy J, Partridge SR, Kemper JA, Braakhuis A, Roy R. Co-design of digital health interventions with young people: A scoping review. Digit HEALTH. 2023;9. 10.1177/20552076231219117. 10.1177/20552076231219117 PMC10722957 38107986

[25] Vargas C, Zorbas C, Longworth GR, Ugalde A, Needham C, Sunil A, Venegas Hargous C, Bennett R, Forrester-Bowling T, Cardoso Richter AP, Allender S. Exploring co-design: a systematic review of concepts, processes, models, and frameworks used in public health research. J Public Health (Oxf). 2025;47(4):e616–39. 10.1093/pubmed/fdaf084 . PMID: 40796276; PMCID: PMC12669994. 40796276 10.1093/pubmed/fdaf084 PMC12669994

[26] Bate P, Robert G. Experience-based design: from redesigning the system around the patient to co-designing services with the patient. Qual Saf Health Care. 2006;15(5):307–10. 10.1136/qshc.2005.016527 . PMID: 17074863; PMCID: PMC2565809. 17074863 10.1136/qshc.2005.016527 PMC2565809

[27] Sanders EBN, Stappers PJ. Co-Creation and the New Landscapes of Design. Co-Design. 2008;4:5–18. 10.1080/15710880701875068.

[28] Greenhalgh T, Jackson C, Shaw S, Janamian T. Achieving Research Impact Through Co-creation in Community-Based Health Services: Literature Review and Case Study. Milbank Q. 2016;94(2):392–429. 10.1111/1468-0009.12197 . PMID: 27265562; PMCID: PMC4911728. 27265562 10.1111/1468-0009.12197 PMC4911728

[29] Langley J, Wolstenholme D, Cooke J. Collective making’ as knowledge mobilisation: the contribution of participatory design in the co-creation of knowledge in healthcare. BMC Health Serv Res. 2018;18:585. 10.1186/s12913-018-3397-y. 30045726 10.1186/s12913-018-3397-y PMC6060522

[30] O’Cathain A, Croot L, Duncan E, Rousseau N, Sworn K, Turner KM, et al. Guidance on how to develop complex interventions to improve health and healthcare. BMJ Open. 2019;9(8). 10.1136/bmjopen-2019-029954. 10.1136/bmjopen-2019-029954 PMC6701588 31420394

[31] Slattery P, Saeri AK, Bragge P. Research co-design in health: a rapid overview of reviews. Health Res Policy Syst. 2020;18(1):17. 10.1186/s12961-020-0528-9 . PMID: 32046728; PMCID: PMC7014755. 32046728 10.1186/s12961-020-0528-9 PMC7014755

[32] Robert G, Locock L, Williams O, Cornwell J, Donetto S, Goodrich J. Co-Producing and Co-Designing. Cambridge University Press; 2022. 10.1017/9781009237024.

[33] AlSwayied G, Frost R, Hamilton FL. Menopause knowledge, attitudes and experiences of women in Saudi Arabia: a qualitative study. BMC Womens Health. 2024;24(1):624. 10.1186/s12905-024-03456-7 . PMID: 39581992; PMCID: PMC11587664. 39581992 10.1186/s12905-024-03456-7 PMC11587664

[34] Bird M, McGillion M, Chambers EM, Dix J, Fajardo CJ, Gilmour M, Levesque K, Lim A, Mierdel S, Ouellette C, Polanski AN, Reaume SV, Whitmore C, Carter N. A generative co-design framework for healthcare innovation: development and application of an end-user engagement framework. Res Involv Engagem. 2021;7(1):12. 10.1186/s40900-021-00252-7 . PMID: 33648588; PMCID: PMC7923456. 33648588 10.1186/s40900-021-00252-7 PMC7923456

[35] Tong A, Sainsbury P, Craig J. Consolidated criteria for reporting qualitative research (COREQ): a 32-item checklist for interviews and focus groups. Int J Qual Health Care. 2007;19(6):349–57. 10.1093/intqhc/mzm042 . Epub 2007 Sep 14. PMID: 17872937. 17872937 10.1093/intqhc/mzm042

[36] Michie S, van Stralen MM, West R. The behaviour change wheel: a new method for characterising and designing behaviour change interventions. Implement Sci. 2011;6:42. 10.1186/1748-5908-6-42 . PMID: 21513547; PMCID: PMC3096582. 21513547 10.1186/1748-5908-6-42 PMC3096582

[37] Michie S, Richardson M, Johnston M, Abraham C, Francis J, Hardeman W, Eccles MP, Cane J, Wood CE. The behavior change technique taxonomy (v1) of 93 hierarchically clustered techniques: building an international consensus for the reporting of behavior change interventions. Ann Behav Med. 2013;46(1):81–95. 10.1007/s12160-013-9486-6. PMID: 23512568. 10.1007/s12160-013-9486-6 23512568

[38] Heaton J, Day J, Britten N. Collaborative research: Building the evidence together. In: Pope C, Mays N, editors. Qualitative research in health care. 4th ed. Hoboken, NJ: Wiley Blackwell; 2024. pp. 197–210.

[39] World Health Organization. WHO guidelines on physical activity and sedentary behaviour. Geneva: World Health Organization. 2020. Available from: https://www.who.int/publications/i/item/9789240015128. Accessed 10 June 2025.

[40] Trischler J, Dietrich T, Rundle-Thiele S. Co-design: from expert- to user-driven ideas in public service design. Public Manag Rev. 2019;21(11):1595–619. 10.1080/14719037.2019.1619810.

[41] Michie S, Atkins L, West R. The behaviour change wheel: a guide to designing interventions. London: Silverback Publishing; 2014.

[42] Braun V, Clarke V. Can I use TA? Should I use TA? Should I not use TA? Comparing reflexive thematic analysis and other pattern-based qualitative analytic approaches. Couns Psychother Res. 2021;21(1):37–47. 10.1002/capr.12360.

[43] Elo S, Kyngäs H. The qualitative content analysis process. J Adv Nurs. 2008;62(1):107 – 15. 10.1111/j.1365-2648.2007.04569.x. PMID: 18352969. 10.1111/j.1365-2648.2007.04569.x 18352969

[44] Schreier M. Qualitative content analysis in practice. London: Sage Publications; 2012. ISBN: 9781849205931.

[45] Mayring P. Qualitative Content Analysis. Forum Qualitative Sozialforschung Forum: Qualitative Social Res. 2000;1(2). 10.17169/fqs-1.2.1089.

[46] Braun V, Clarke V. Using thematic analysis in psychology. Qual Res Psychol. 2006;3(2):77–101. 10.1191/1478088706qp063oa.

[47] Casey M, Dabkowski E, de Gracia MRL, Moore KA, Kennedy GA, Porter JE, Nasstasia Y, Alvarenga ME. Socioecological factors influencing physical activity engagement for women across the menopausal transition: a systematic review. Menopause. 2024;31(5):433–46. Epub 2024 Mar 9. PMID: 38595173. 38595173 10.1097/GME.0000000000002337

[48] Haynes A, Wallbank G, Gilchrist H, Sherrington C, West CA, Oliveira JS, O’Rourke S, Tiedemann A. What do older women want from a physical activity program? Stakeholder consultation to optimise design and recruitment for the Active Women over 50 trial. BMC Public Health. 2024;24(1):2920. 10.1186/s12889-024-20345-8 . PMID: 39438858; PMCID: PMC11494785. 39438858 10.1186/s12889-024-20345-8 PMC11494785

[49] Wallbank G, Haynes A, Tiedemann A, et al. Designing physical activity interventions for women aged 50+: a qualitative study of participant perspectives. BMC Public Health. 2022;22:1855. 10.1186/s12889-022-14237-y. 36195939 10.1186/s12889-022-14237-y PMC9531643

[50] Walsh A, Simpson EEA. Health cognitions mediate physical (in)activity and walking in midlife women. Maturitas. 2020;131:14–20. 10.1016/j.maturitas.2019.10.005 . Epub 2019 Oct 16. PMID: 31787142. 31787142 10.1016/j.maturitas.2019.10.005

[51] Murtagh MJ, Hepworth J. Narrative review of changing medical and feminist perspectives on menopause: From femininity and ageing to risk and choice. Psychol Health Med. 2005;10(3):276–90. 10.1080/13548500500093225.

[52] Godoy-Izquierdo D, de Teresa C, Mendoza N. Exercise for peri- and postmenopausal women: Recommendations from synergistic alliances of women’s medicine and health psychology for the promotion of an active lifestyle. Maturitas. 2024;185:107924. 10.1016/j.maturitas.2024.107924 . Epub 2024 Jan 28. PMID: 38599003. 38599003 10.1016/j.maturitas.2024.107924

[53] Sheen F, Porter L, Papakonstantinou T, Ceka M, Bondaronek P. Living well? The unintended consequences of highly popular commercial fitness apps through social listening using Machine-Assisted Topic Analysis: Evidence from X. Br J Health Psychol. 2025;30(4):e70026. 10.1111/bjhp.70026 . PMID: 41121969; PMCID: PMC12541294. 41121969 10.1111/bjhp.70026 PMC12541294

[54] Izquierdo M, de Souto Barreto P, Arai H, Bischoff-Ferrari HA, Cadore EL, Cesari M, Chen LK, Coen PM, Courneya KS, Duque G, Ferrucci L, Fielding RA, García-Hermoso A, Gutiérrez-Robledo LM, Harridge SDR, Kirk B, Kritchevsky S, Landi F, Lazarus N, Liu-Ambrose T, Marzetti E, Merchant RA, Morley JE, Pitkälä KH, Ramírez-Vélez R, Rodriguez-Mañas L, Rolland Y, Ruiz JG, de Sáez ML, Villareal DT, Waters DL, Won Won C, Vellas B, Fiatarone Singh MA. Global consensus on optimal exercise recommendations for enhancing healthy longevity in older adults (ICFSR). J Nutr Health Aging. 2025;29(1):100401. 10.1016/j.jnha.2024.100401 . Epub 2025 Jan 1. PMID: 39743381; PMCID: PMC11812118. 39743381 10.1016/j.jnha.2024.100401 PMC11812118

[55] Adams M, Gordt-Oesterwind K, Bongartz M, Zimmermann S, Seide S, Braun V, Schwenk M. Effects of Physical Activity Interventions on Strength, Balance and Falls in Middle-Aged Adults: A Systematic Review and Meta-Analysis. Sports Med Open. 2023;9(1):61. 10.1186/s40798-023-00606-3 . PMID: 37466877; PMCID: PMC10356733. 37466877 10.1186/s40798-023-00606-3 PMC10356733

[56] Vasudevan A, Ford EM. Factors and Barriers Towards Initiating and Maintaining Strength Training in Women: a Systematic Review and Meta-synthesis. Prev Sci. 2022;23:674–95. 10.1007/s11121-021-01328-2. 34800250 10.1007/s11121-021-01328-2 PMC9072266

[57] McIntosh E, Horspool M, Levesley M, Logan P, Klonizakis M. The co-design of an exercise-based, lifestyle intervention for people with venous leg ulcers; a self-care, expert-supported strategy for a chronic condition. Int Wound J. 2023;20(7):2528–39. 10.1111/iwj.14117 . Epub 2023 Mar 8. PMID: 36883381; PMCID: PMC10410337. 36883381 10.1111/iwj.14117 PMC10410337

[58] Brawley LR, Rejeski WJ, King AC. Promoting physical activity for older adults: the challenges for changing behavior. Am J Prev Med. 2003;25(3 Suppl 2):172 – 83. 10.1016/s0749-3797(03)00182-x. PMID: 14552942. 10.1016/s0749-3797(03)00182-x 14552942

[59] Hill KD, Hunter SW, Batchelor FA, Cavalheri V, Burton E. Individualized home-based exercise programs for older people to reduce falls and improve physical performance: A systematic review and meta-analysis. Maturitas. 2015;82(1):72–84. Epub 2015 Apr 29. PMID: 25989701. 25989701 10.1016/j.maturitas.2015.04.005

[60] Halperin I, Wulf G, Vigotsky AD, Schoenfeld BJ, Behm DG. Autonomy: A Missing Ingredient of a Successful Program? Strength Conditioning J. August 2018;40(4):18–25. 10.1519/SSC.0000000000000383.

[61] Baretta D, Perski O, Steca P. Exploring Users’ Experiences of the Uptake and Adoption of Physical Activity Apps: Longitudinal Qualitative Study. JMIR Mhealth Uhealth. 2019;7(2):e11636. 10.2196/11636PMID: 30735143PMCID: 6384536. 30735143 10.2196/11636 PMC6384536

[62] D’Addario M, Baretta D, Zanatta F, Greco A, Steca P. Engagement Features in Physical Activity Smartphone Apps: Focus Group Study With Sedentary People. JMIR Mhealth Uhealth. 2020;8(11):e20460. 10.2196/20460 . PMID: 33196450; PMCID: PMC7704278. 33196450 10.2196/20460 PMC7704278

[63] Joseph RP, Keller C, Vega-López S, Adams MA, English R, Hollingshead K, Hooker SP, Todd M, Gaesser GA, Ainsworth BE. A Culturally Relevant Smartphone-Delivered Physical Activity Intervention for African American Women: Development and Initial Usability Tests of Smart Walk. JMIR Mhealth Uhealth. 2020;8(3):e15346. 10.2196/15346 PMID: 32130198 PMCID: 7076402. 32130198 10.2196/15346 PMC7076402

[64] Joseph RP, Todd M, Ainsworth BE, Vega-López S, Adams MA, Hollingshead K, Hooker SP, Gaesser GA, Keller C. Smart Walk: A Culturally Tailored Smartphone-Delivered Physical Activity Intervention for Cardiometabolic Risk Reduction among African American Women. Int J Environ Res Public Health. 2023;20(2):1000. 10.3390/ijerph20021000 . PMID: 36673756; PMCID: PMC9859082. 36673756 10.3390/ijerph20021000 PMC9859082

[65] El Masri A, Kolt GS, George ES. Physical activity interventions among culturally and linguistically diverse populations: a systematic review. Ethn Health. 2022;27(1):40–60. Epub 2019 Aug 26. PMID: 31446773. 31446773 10.1080/13557858.2019.1658183

[66] Resnicow K, Baranowski T, Ahluwalia JS, Braithwaite RL. Cultural sensitivity in public health: defined and demystified. Ethn Dis. 1999 Winter;9(1):10–21. PMID: 10355471. 10355471

[67] El Masri A, Kolt GS, George ES. Feasibility and acceptability of a culturally tailored physical activity intervention for Arab-Australian women. BMC Womens Health. 2021;21:131. 10.1186/s12905-021-01250-3. 33784997 10.1186/s12905-021-01250-3 PMC8008684

[68] Alzahrani AA, Gelius P, Bauman AE, Gebel K. Physical activity policies in Saudi Arabia and Oman: a qualitative study using stakeholder interviews. Health Res Policy Syst. 2024;22(1):111. 10.1186/s12961-024-01192-w . PMID: 39160530; PMCID: PMC11331687. 39160530 10.1186/s12961-024-01192-w PMC11331687

[69] Greenhalgh T, Stramer K, Bratan T, Byrne E, Russell J, Potts H. Adoption and Non-Adoption of a Shared Electronic Summary Record in England: a Mixed-Method Case Study. BMJ. (Clinical Res Ed). 2010;340(7761):c3111. 10.1136/bmj.c3111 20554687

[70] Shaw JA, Donia J. The Sociotechnical Ethics of Digital Health: A Critique and Extension of Approaches From Bioethics. Front Digit Health. 2021;3:725088. 10.3389/fdgth.2021.725088 . PMID: 34713196; PMCID: PMC8521799. 34713196 10.3389/fdgth.2021.725088 PMC8521799

[71] Brooks BA, Davis S, Frank-Lightfoot L, Kulbok PA, Poree S, Sgarlata L. (2014, 2018). Building a Community Health Worker Program: The Key to Better Care, Better Outcomes, & Lower Costs. Published by CommunityHealth Works. Chicago: Authors.

[72] Casillas A, Abhat A, The Los Angeles County Department of Health Services Health Technology Navigators. A novel health workforce to digitally empower patient communities in safety net systems. J Med Access. 2024;8:27550834231223024. PMID: 38225933; PMCID: PMC10788075. 38225933 10.1177/27550834231223024 PMC10788075

[73] Harrison R, Walton M, Chitkara U, Manias E, Chauhan A, Latanik M, Leone D. Beyond translation: Engaging with culturally and linguistically diverse consumers. Health Expect. 2020;23(1):159–68. 10.1111/hex.12984 . Epub 2019 Oct 18. PMID: 31625264; PMCID: PMC6978859. 31625264 10.1111/hex.12984 PMC6978859

[74] Moll S, Wyndham-West M, Mulvale G, et al. Are you really doing ‘codesign’? Critical reflections when working with vulnerable populations. BMJ Open. 2020;10:e038339. 10.1136/bmjopen-2020-038339. 33148733 10.1136/bmjopen-2020-038339 PMC7640510

[75] Chauhan A, Leefe J, Shé ÉN, et al. Optimising co-design with ethnic minority consumers. Int J Equity Health. 2021;20:240. 10.1186/s12939-021-01579-z. 34736455 10.1186/s12939-021-01579-z PMC8567634

[76] O’Brien N, Heaven B, Teal G, Evans EH, Cleland C, Moffatt S, Sniehotta FF, White M, Mathers JC, Moynihan P. Integrating Evidence From Systematic Reviews, Qualitative Research, and Expert Knowledge Using Co-Design Techniques to Develop a Web-Based Intervention for People in the Retirement Transition. J Med Internet Res. 2016;18(8):e210. 10.2196/jmir.5790 . PMID: 27489143; PMCID: PMC4989122. 27489143 10.2196/jmir.5790 PMC4989122

[77] Zhou T, Kirby-Ginns S, Salman D, et al. Using codesign workshops to develop a conceptual framework for a mobile health app targeting chronic low back pain self-management. BMJ Open. 2025;15:e093386. 10.1136/bmjopen-2024-093386. 39987005 10.1136/bmjopen-2024-093386 PMC11848688

[78] Lowy A, Hood P. The power of the 2 x 2 matrix: using 2 x 2 thinking to solve business problems and make better decisions. San Francisco: Jossey-Bass; 2004.

[79] Piquer-Martinez C, Urionagüena A, Benrimoj SI, Calvo B, García-Cárdenas V, Amador-Fernandez N, Gastelurrutia MA, Martinez Martinez F. Strategic interventions and a novel model for the integration of community pharmacy and primary care in Spain: qualitative insights. BMJ Open. 2024;14(12):e086285. 10.1136/bmjopen-2024-086285 . PMID: 39732484; PMCID: PMC11684000. 39732484 10.1136/bmjopen-2024-086285 PMC11684000

[80] Alharbi BFH, Baker P, Pavey T, Alharbi MF. Investigating the beliefs of Saudi females regarding physical activity: A qualitative exploration. Int J Qualitative Stud Health Well-Being. 2024;19(1):2296696. 10.1080/17482631.2023.2296696. 10.1080/17482631.2023.2296696 PMC10763862 38127866

[81] Dehling T, Gao F, Schneider S, Sunyaev A. Exploring the far side of mobile health: information security and privacy of mobile health apps on iOS and Android. JMIR mHealth uHealth. 2015;3(1). 10.2196/mhealth.3672. 10.2196/mhealth.3672 PMC4319144 25599627

[82] Villa-García L, Davey V, Peréz LM, Soto-Bagaria L, Risco E, Díaz P, Kuluski K, Giné-Garriga M, Castellano-Tejedor C, Inzitari M. Co-designing implementation strategies to promote remote physical activity programs in frail older community-dwellers. Front Public Health. 2023;11:1062843. 10.3389/fpubh.2023.1062843 . PMID: 36960372; PMCID: PMC10028273. 36960372 10.3389/fpubh.2023.1062843 PMC10028273

[83] Skivington K, Matthews L, Simpson SA, Craig P, Baird J, Blazeby JM, et al. A new framework for developing and evaluating complex interventions: update of Medical Research Council guidance. BMJ. 2021. 10.1136/bmj.n2061. 374. 34593508 10.1136/bmj.n2061 PMC8482308

[84] Marques MM, Wright AJ, Corker E, Johnston M, West R, Hastings J, Zhang L, Michie S. The Behaviour Change Technique Ontology: Transforming the Behaviour Change Technique Taxonomy v1. Wellcome Open Res. 2024;8:308. 10.12688/wellcomeopenres.19363.2. 37593567 10.12688/wellcomeopenres.19363.2 PMC10427801

[85] Yardley L, Morrison L, Bradbury K, Muller I. The Person-Based Approach to Intervention Development: Application to Digital Health-Related Behavior Change Interventions. J Med Internet Res. 2015;17(1):e30. 10.2196/jmir.4055. 25639757 10.2196/jmir.4055 PMC4327440

[86] May CR, Cummings A, Girling M, Bracher M, Mair FS, May CM, Murray E, Myall M, Rapley T, Finch T. Using Normalization Process Theory in feasibility studies and process evaluations of complex healthcare interventions: a systematic review. Implement Sci. 2018;13(1):80. 10.1186/s13012-018-0758-1 . PMID: 29879986; PMCID: PMC5992634. 29879986 10.1186/s13012-018-0758-1 PMC5992634

[87] Alharbi MF. An analysis of the Saudi health-care system’s readiness to change in the context of the Saudi National Health-care Plan in Vision 2030. Int J Health Sci (Qassim). 2018 May-Jun;12(3):83–7. PMID: 29896076; PMCID: PMC5969787. PMC5969787 29896076

[88] Suleiman AK, Ming LC. Transforming healthcare: Saudi Arabia’s vision 2030 healthcare model. J Pharm Policy Pract. 2025;18(1):2449051. 10.1080/20523211.2024.2449051 . PMID: 39845746; PMCID: PMC11753010. 39845746 10.1080/20523211.2024.2449051 PMC11753010

