# Supplementary material for: Co-designing a culturally appropriate mHealth physical activity intervention for midlife women experiencing menopause in Saudi Arabia: stakeholder recommendations
Source: BMC Public Health. 2026 May 27;26:2018. doi: 10.1186/s12889-026-27624-6 (PMC13325748; doi:10.1186/s12889-026-27624-6)
Supplement: Supplementary file 1 — Supplementary Material 1. [file 12889_2026_27624_MOESM1_ESM.docx]

**Co-design Workshops Topic Guide (semi-structured)**

Co-develop actionable recommendations (and design principles) for tailoring messages and optimising mobile PA interventions for midlife women undergoing menopause in Saudi Arabia: using co-design workshop methods.

| #WS | Who (stakeholders) | Why  (main objectives) | Topics for discussion | Time allocated | Expected output(s) |
| --- | --- | --- | --- | --- | --- |
| **WS 1**  (empathy & co-ideation)  60- 100 min | **End-user**  A group of potential end-users from menopausal women who were recruited from the earlier interviews study.  physically inactive  * Invite 8 for the session. | Intro + icebreaker | | 10 min |  |
|  |  | 1. Reflect and gain feedback on the main themes generated from the interview study with potential end-users to check and refine findings. | Present the main relevant findings from the previous study.  Use Figure 1 to summarise the identified main characteristics needed in the mobile PA interventions to be considered suitable and motivational for women undergoing menopause. | 15 min | Checking and refining findings and proposed recommendations. |
|  |  |  | Activity 1: Addressing menopausal relevance / needs  How should the PA intervention/app acknowledge the menopause experience in its content to ensure its relevance in bringing about desired outcomes in menopausal women? | 25 min |  |
|  |  | Break | | 10 min |  |
|  |  | 2. Brainstorm practical strategies to overcome the contextual and motivational barriers to PA among menopausal women in Saudi Arabia | Activity 2: Overcoming barriers  Use storyboards of anonymised women’s narratives to describe menopausal women’s lived experiences (user needs) according to the themes generated from the interviews study. Graphics present existing challenges (barriers) encountered in relation to acceptability, accessibility, feasibility, and ways for fitting exercise into menopausal women’s lives. Each storyboard will introduce a real-based scenario (a unique character/setting/contextual barrier to PA) and possible solutions.   - What areas/ strategies should the intervention focus on consider addressing women's needs (common contextual barriers identified) and motivate them to exercise during menopause?  1. Busy schedule: 2. Target women’s self-efficacy and overcome their fear of injury associated with exercise. 3. Normalise fatigue and work to improve muscle strength. | 40 min | Purpose for the intervention - Identify **appropriate intervention types** (e.g., educational, enablement, habit formation, and engagement…) to address identified PA needs among menopausal women.  - **Tailor strategies to needs:** Justify each selected intervention type based on the specific PA challenges faced by menopausal women. |
|  |  |  | How do we ensure respect and alignment with the local culture while ensuring the effectiveness of exercise delivery?  How much human interactions need to be in there? and how? | 10 min | Ensuring strategies resonate culturally and practically. |
| **WS 2/3/4**  (empathy & co-ideation)  90- 120 min max | Experts’ sessions  + HCPs: GPs, Gynecologists, health educators, physiotherapists, psychologists  + fitness trainers  * Invite 7-8 for each session (as possible). | Identify and refine who we should target? what the intervention should do (content) and how it should do it (behavioural functions). | Visualise the user needs using the storyboards and present the main relevant findings from the previous end-user session, and ask the experts for feedback, agreement, disagreement, and refinement. | 30 min | Refined the purpose for the intervention/ its strategies (following insights and inputs from different stakeholders) |
|  |  | Part 1: focuses on the educational needs (messages and topics) that should be incorporated that are relevant and useful.  Identify, refine, and prioritise. | Activity 1: What the intervention (app) should do? What content to cover?   - Which information (content) should be incorporated in the digital platform or app targeting PA for menopausal women? - From whom should and should not be delivered? - Who should be involved in the content creation process? - How much PA is needed (intensity/ duration/ frequency) - What types of exercises do women need to prioritise at menopause to receive expected benefits? - What type of exercise activity should we focus on? Whether focusing on walking vs muscle strengthening exercises vs combination of different activities? Why?   - Any menopause information needs to be covered? Easy to digest?  e.g.,   - What is Menopause? - Common menopausal symptoms? - Its impact on everyday life? - What can we do about it? - Perceived benefits of PA on menopause and improved QoL? | 50 min | Iterative list of proposed intervention content  **- Outline suggested topics**: (most/least important).  - C**ore messages**: prioritise key educational messages. Emphasise those messages most likely to resonate with and empower Saudi women in menopause. |
|  |  | Break | | 10 min |  |
|  |  | Part 2: focuses on the behavioural strategies (how the structure and content of the intervention can facilitate or inhibit PA behavioural change). | Activity 2: How should the intervention do it? (behavioural functions)   - How and what behavioural strategies do you think the PA digital intervention (or app) should include to address needs and motivate end users in bringing about desired PA outcomes? - The most useful and least useful functions? - To what extent, do you think it is important for menopausal women to have a supportive community (social support function) to share their menopause and exercise experience and motivate each other? And how can social support be offered? - How about feedback/ coaching from experts? from whom specifically? (Feedback on performance) - How much human interactions need to be in there? and how? | 50 min | Suggested behavioural strategies to embed. |
| **WS 5** Feasibility and implementation  90- 120 min | Cross-sectoral  +Policymakers  + tech experts (app designers)  * Invite 4 for each session (if possible). | Focuses on the feasibility of the design features and implementation options inc. delivery of the messages/ intervention and integration with existing systems. | Present a brief recap of outcomes from the previous sessions.  - Activity 1: What are the most important promotional messages/ intervention components to prioritise? | 15 min | Feasibility insights on potential intervention strategies, features, and implementation strategies  - **Prioritise behavioural strategies**: Categorise into essential, useful, and optional based on different stakeholders’ feedback.  - **Assess feasibility**: Identify which strategies can realistically be implemented first based on practicality and cultural appropriateness.  **- Assess Implementation Strategies**: consider the logistics of delivering the app, ensuring accessibility, affordability, and possible integration with local healthcare systems for broader support and reinforcement. |
|  |  |  | - Activity 2: co-prioritisation activity: rank the potential design principles.   - How should the high-priority functionalities of a menopause-friendly PA app be translated into design-specific features? - Best desirable/ feasible way for a demonstration of exercises. - How do we ensure respect and alignment with the local culture while ensuring the effectiveness of exercise delivery (of real women)? - How feasible is it to embed social features to connect with other users? Connect with experts? | 30 min |  |
|  |  |  | Break | 10 min |  |
|  |  |  | How should they be delivered? validated? and implemented?   - Accessibility and affordability - Cost-wise? price-sensitive - What would be the best business model: - (Affordable for users, built on a sustainable financial model) - Free of charge (e.g., gov. funding) / subscription/ in-app purchases/ freemium model? | 15 min |  |
|  |  |  | Delivery and integration strategies   - How do we intend to this intervention to fit with the health services in Saudi? - Dissemination channels? Supported by formal entities MOH / SPA or commercially/ publicly available on the market? - Mode of delivery: off-line/ live sessions/ facilitated (supervised) or unsupervised. - How can we ensure the accessibility of a remote (mobile) PA intervention for menopausal women in Saudi Arabia? - Entirely remote-based intervention? - What support is needed to ensure inclusivity of women with low digital literacy? - How to ensure the inclusivity of women with low digital literacy? | 15 min |  |


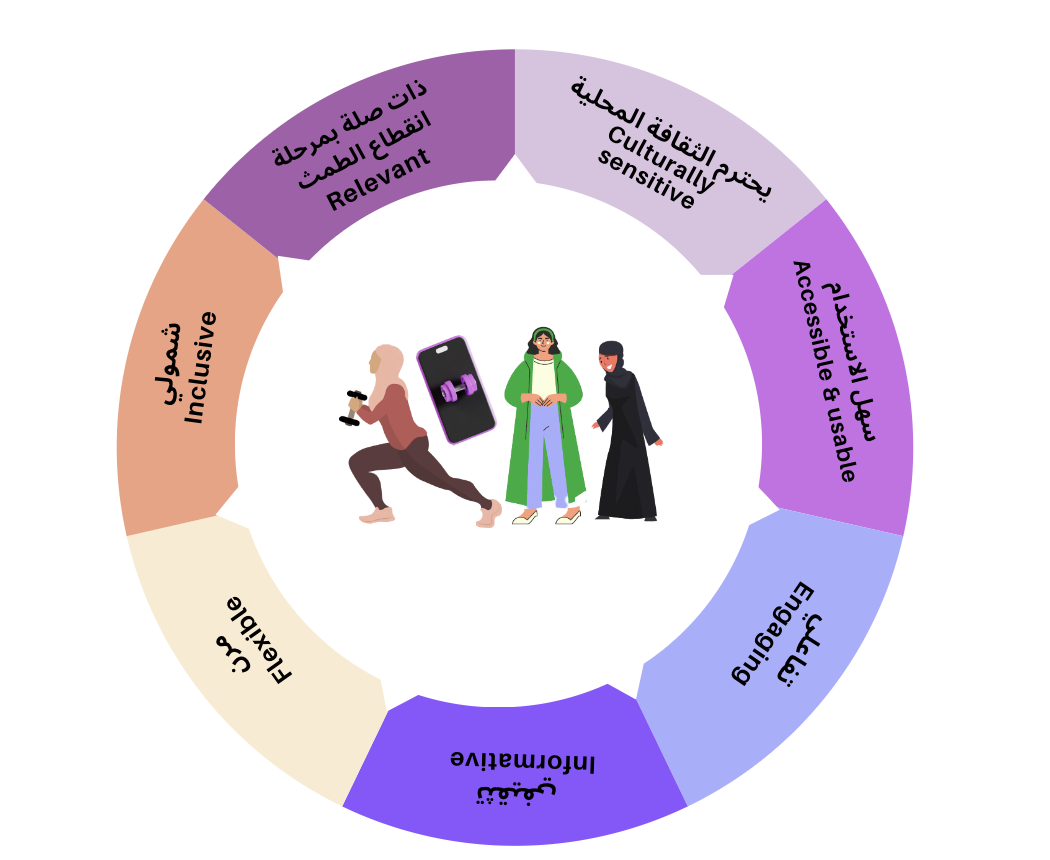


Figure 1: Women’s perspectives and preferences regarding design prinicples (extracted from the prior qualitative interview with midlife Saudi women).
