# Supplementary material for: Co-designing a culturally appropriate mHealth physical activity intervention for midlife women experiencing menopause in Saudi Arabia: stakeholder recommendations
Source: BMC Public Health. 2026 May 27;26:2018. doi: 10.1186/s12889-026-27624-6 (PMC13325748; doi:10.1186/s12889-026-27624-6)
Supplement: Supplementary file 2 — Supplementary Material 2. [file 12889_2026_27624_MOESM2_ESM.docx]

**Supplementary figure 2. The outputs of the feasibility prioritisation activity conducted with multiple stakeholders in Workshop 5.**

**
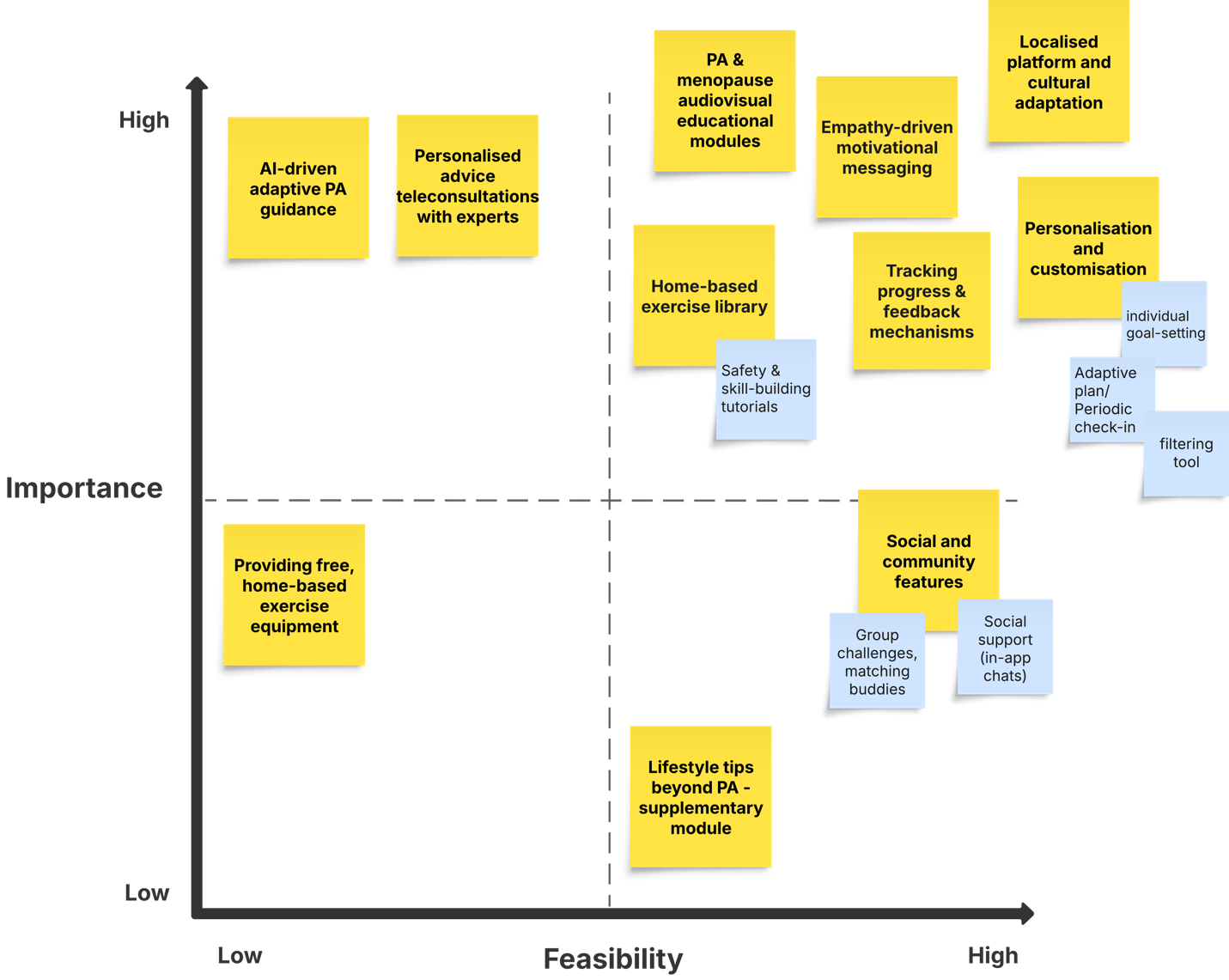
**

**Do Now ^a^**

**Maybe Later ^c^**

**Do Next ^b^**

**Don’t Do ^d^**

_a: Ranked by most stakeholders as both high-impact and highly feasible, recommended for immediate implementation.
b: Ranked as both moderate-impact and highly feasible, recommended for mid-term implementation.
c: Ranked as both high-impact but moderate or less feasible, recommended for future exploration.
d: Ranked as both less-impactful but moderate or less feasible, not recommended._
