# Supplementary material for: Co-designing a culturally appropriate mHealth physical activity intervention for midlife women experiencing menopause in Saudi Arabia: stakeholder recommendations
Source: BMC Public Health. 2026 May 27;26:2018. doi: 10.1186/s12889-026-27624-6 (PMC13325748; doi:10.1186/s12889-026-27624-6)
Supplement: Supplementary file 3 — Supplementary Material 3. [file 12889_2026_27624_MOESM3_ESM.docx]

**Supplementary file 3:**

Table of theoretical mapping linking the stakeholder co-designed, prioritised intervention recommendations with corresponding COM-B, TDF, and BCW

| Co-designed  intervention design recommendations | Example of stakeholder input | COM-B primary  targets | TDF domains | BCW intervention functions | Interpretation of plausible behavioural mechanisms | | Assumptions |
| --- | --- | --- | --- | --- | --- | --- | --- |
|  |  |  |  |  | Primary pathway | Indirect pathway |  |
| Tailored educational modules on PA and menopause | *“Most of us don’t really know where to begin with physical activity or what exercises are safe for us. If we had tailored guides on what’s appropriate or beneficial for us, I suppose it would be much easier to start”* Stakeholder 3, Menopausal Women, WS1  *"I imagine the content of a PA app to be adapted… That means it would recognise menopausal symptoms in its content to help women feel more empowered"* Stakeholder 20, Fitness Trainer, WS4 | Psychological capability;  Reflective motivation | Knowledge;  Skills;  Beliefs about consequences | Education;  Enablement;  Persuasion | **Psychological capability** →  via knowledge acquisition and skill-building (MoA), enabling women to understand safe and effective PA during menopause  **Reflective motivation** → via beliefs about consequences (MoA), by linking PA to menopause symptom relief, wellbeing, and independence, and identity alignment (MoA), with women’s lived experiences to provide emotionally resonant and meaningful reasons to prioritise PA during menopausal transition | **Physical capability →** via self-efficacy enhancement and reduced fear of injury (MoA), building confidence to try new exercise forms (e.g., resistance training)  **Automatic motivation →**  via repeated exposure to consistent, accessible PA guidance (MoA), which may reinforce habit formation initiation and normalising PA routines over time | • Menopause-specific content is more compelling than generic PA advice  • Safety concerns are primary barriers to PA initiation • Educational content will be literacy appropriate and culturally acceptable |
| Home-based, adaptable, structured exercise library  confidence-building tutorials | *“The app should have a pre-beginner level exercise bundle for us, those who’ve never exercised”* Stakeholder 2, Menopausal Woman, WS1 | Physical capability; Psychological capability;  Physical opportunity | Skills;  Environmental context;  Beliefs about capabilities;  Emotion | Training; Enablement;  Environmental Restructuring | **Physical capability** → via progressive skill acquisition and graded task mastery (MoA), building competence through structured pre-beginner to advanced tutorials  **Psychological capability** → via self-efficacy enhancement and fear reduction (MoA), step by step, structured progression builds confidence and reduces exercise anxiety  **Physical opportunity** → via environmental restructuring (MoA), removing barriers related to cost, transport, modesty, and facility access through home-based delivery | **Reflective motivation** → via beliefs about capabilities (MoA), as skills develop, motivation to sustain PA could be reinforced  **Automatic motivation** → via habit formation and routine initiation (MoA), repeated engagement with home-based sessions may gradually normalises PA within women’s routines   **Social opportunity** →  via family approval and indirect support (MoA), as home-based PA may encourage tacit acceptance or encouragement from household members, though this was **absent** in co-design discussions | • Women have adequate private home spaces for exercise • Video tutorials can effectively teach movement without in-person instruction |
| Empathy-driven messaging | *“It's natural for a woman to see her routine change completely with the menopausal transition. Connecting PA with self-care, purpose, and self-worth can be more encouraging than focusing on aesthetics or weight loss”* Stakeholder 12, Clinical Psychologist, WS3 | Reflective motivation | Emotion;  Social role and Identity;  Beliefs about Consequences | Persuasion;  Modelling;  Enablement | **Reflective motivation** → via values realignment and identity reframing (MoA), positioning PA as self-care and resilience rather than appearance or weight loss focused   **Reflective motivation** → via autonomous motivation and value alignment (MoA), messaging links PA to women’s lived values and needs (e.g., wellbeing, emotional relief, family functioning), and via emotional regulation, reducing guilt about self-investment and legitimising personal PA time | **Social opportunity →** via normative influence and stigma reduction (MoA), empathetic messaging that aligns with cultural values (self-care enables family care) supports collective acceptance  **Psychological capability →** via belief restructuring (MoA), challenging generic PA messaging about exercise purposes (e.g., exercise is only for weight loss) may complement educational content | • Self-care messaging resonates with Saudi women's values • Guilt about self-investment is a significant barrier • Value-based intrinsic motivation is more sustainable than appearance-based motivation |
| Progress tracking and feedback mechanisms | *“Prompts on Immediate benefits like feeling energised, or gaining self-control post-workout are often underrated but essential for sustained motivation”* Stakeholder 20, Fitness Trainer, WS4 | Reflective motivation | Goals; Reinforcement Behavioural regulation | Incentivisation;  Enablement;  Persuasion | **Reflective motivation** → via immediate reinforcement loops and self-regulation enhancement (MoA), emphasising immediate psychological benefits (energy, mood, control) to strengthen beliefs about PA value and sustain engagement motivation | **Automatic motivation** → via positive reinforcement and associative learning (MoA), regular feedback creates positive PA associations with immediate psychological rewards and possibly supports habit formation  **Psychological capability** → via self-awareness development and goal-setting skills (MoA), progress feedback enhances behavioural regulation capabilities | • Immediate benefits are noticeable and valued.  • Progress tracking motivates rather than creates pressure • Feedback timing aligns with natural PA patterns |
| Personalisation and customisation | *“It is important to offer personalised exercise plans... Initial assessment can create individualised plans based on a woman’s specific profile and physical abilities”* Stakeholder 20, Fitness trainer, WS4  *“It is important to follow up the user and adapt exercise recommendations according to women’s existing health. If certain workouts aren’t suitable anymore, alternatives like using low-impact exercise machines can be suggested then"* Stakeholder 4, Gynaecologist, WS2 | Reflective motivation; Psychological capability; Physical capability | Skills;  Beliefs about Capabilities;  Beliefs about Consequences  Goals; Behavioural Regulation; Memory, Attention, and Decision Processes | **Enablement**;  **Education**; **Training** | **Reflective motivation →** via relevance optimisation and goal alignment (MoA), individualised plans make PA feel achievable and personally meaningful  **Psychological capability →** via *tailored* assessment and cognitive load reduction (MoA), personalised recommendations provide clarity and improve understanding of suitable PA options  **Physical capability →** via skill-appropriate matching and progression adaptation (MoA), ensuring exercises match individual capabilities and health limitations | **Physical opportunity →** via adaptive accommodation and accessibility maintenance (MoA), providing alternative options for physical limitations or health changes maintains feasible PA access  **Automatic motivation** → via *reduced* decision fatigue (MoA), personalised guidance may simplify daily PA decisions | • Women will engage honestly with assessment processes  • Personalised content increases adherence more than generic approaches |
| Social and community support features | *"I think adding a support group function could really help. First, it would provide emotional and social support for menopausal women who don’t have it.. It’s not just me, others are going through the same thing too!"* Stakeholder 12, Clinical psychologist, HCP, SW3 | Social opportunity; Reflective motivation | Social influences; ReinforcementSocial/ professional role and identity | Social support; Enablement;  Modelling; Persuasion; Education;  Incentivisation | **Social opportunity →** via peer modelling and collective belonging (MoA), connecting women with similar experiences normalises menopause PA and provides ongoing emotional support and accountability  **Reflective motivation →** via social reinforcement and identity validation (MoA), seeing others "like them" succeed strengthens personal relevance and positive beliefs about PA capability | **Psychological capability →** via collective knowledge sharing and peer problem-solving (MoA), community exchange enhances practical PA skills and barrier management strategies (e.g., how others manage barriers, practical tips), indirectly building capability  . | • Women desire peer connection around menopause experiences • Social proof is influential in this cultural context • Online communities can create genuine supportive relationships • Peer support is culturally acceptable for Saudi women |
| Cultural tailoring *(interface and messaging)* | *“Aligning the intervention to the local culture should be a top priority from the start. Resonating PA messages with Islamic practices would be a strong motivator for middle-aged Saudi women”*  Stakeholder 22, Policymaker, WS5 | Social opportunity; Reflective motivation | Social influences;  Environmental context and resources; Social role and identity; Beliefs about Consequences | Environmental Restructuring; Education;  Persuasion; Enablement | **Social opportunity →** via cultural coherence and stigma reduction (MoA), embedding Islamic values and Arabic language increases acceptability and creates enabling social conditions  **Reflective motivation →** via values alignment and identity integration (MoA), linking PA to Islamic concepts (linking PA to comfort in prayer) and framing menopause as renewal instead of age of despair) provide meaningful, faith-consistent reasons to engage | **Automatic motivation →** via cultural cue integration and positive associations (MoA), repeated exposure to familiar Islamic framings may create natural emotional connections to PA  **Psychological capability →** via cognitive familiarity and comprehension enhancement (MoA), Arabic language and cultural references improve understanding and reduce processing barriers  **Physical opportunity →** via modesty accommodation and privacy-by-design (MoA), culturally appropriate formats may indirectly reduce environmental barriers, enabling culturally appropriate exercise contexts | • Islamic values can be authentically aligned with PA promotion • Religious framing enhances rather than restricts motivation |

Abbreviation: MoA= mechanism of action

Table of theoretical mapping linking the stakeholder co-designed supporting strategies with corresponding COM-B, TDF, and BCW

| Co-designed supporting strategies | Example of stakeholder input | COM-B primary  targets | TDF domains | BCW intervention functions | BCW policy category | Interpretation of plausible behavioural mechanisms | | Assumptions |
| --- | --- | --- | --- | --- | --- | --- | --- | --- |
|  |  |  |  |  |  | Primary pathway | Indirect pathway |  |
| Technical support and training | *“In my opinion, providing technical support within the app or through external resources would be valuable. I envision engaging the community by creating opportunities for young women to volunteer as tech ambassadors or encouraging users to seek support from family members, such as a daughter or even a granddaughter”* Stakeholder 23, App Developer, WS5 | Psychological capability; Physical opportunity | Skills;  Environmental Context and Resources;  Memory; Attention, and Decision Processes | Training;  Enablement; Environmental Restructuring | Service Provision | **Psychological capability →** via digital skill acquisition and confidence building (MoA), providing onboarding tutorials and troubleshooting resources to enhance women's ability to navigate digital tools effectively  **Physical opportunity →** via barrier removal and access facilitation (MoA), reducing technical obstacles through comprehensive support systems that ensure practical app usability | **Social opportunity →** via intergenerational support activation and family engagement (MoA), leveraging daughters/granddaughters as tech ambassadors creates enabling social environments for digital adoption  **Reflective motivation →** via self-efficacy enhancement and competence beliefs (MoA), successful digital navigation strengthens confidence in technology use and sustained engagement motivation | • Women are willing to ask family members for technical help • Intergenerational support is culturally acceptable and available  • Technical confidence translates to sustained app usage |
| Healthcare integration and HCPs involvement | *“Developing content with input from Saudi-based healthcare professionals, like doctors and exercise and behavioural experts can enhance evidence base and increase a sense of ownership among Saudi healthcare providers”* Stakeholder 22, Policymaker, WS5  *“If my doctor knows I’ve made progress on my PA over the last three months, possibly by integrating my data with the Sehhaty app, it could make routine consultations with my GP more productive.”* – Stakeholder 1, menopausal woman, WS5 | Reflective motivation;  Social opportunity | Knowledge; Social/Professional Role and Identity; Beliefs about Capabilities and Consequences; Environmental Context and Resources. | Education;  Persuasion;  Social Support (professional) | Guidelines and Communication/ Marketing; Environmental Planning | **Reflective motivation** → via professional validation and clinical credibility (MoA), healthcare provider endorsement reinforces beliefs that PA is beneficial during menopause  **Social opportunity** → via trusted authority influence and accountability creation (MoA), healthcare involvement provides professional support that enables sustained PA engagement | **Psychological capability →** via expert knowledge integration and contextual guidance (MoA), Saudi healthcare professional input ensures culturally and literacy appropriate, evidence-based content that enhances understanding  **Physical opportunity** → via healthcare system integration and routine care embedding (MoA), connection with national platforms (Sehhaty) or primary care pathways structurally could enable access and continuity  **Automatic motivation →** via routine integration of sharing progress with providers may normalise PA tracking and embed it as a habitual part of care | • Women trust and value healthcare provider recommendations  • Integration with Sehhaty app is technically feasible and acceptable • Professional endorsement increases rather than medicalises PA engagement during menopause |
| Cross-sector partnerships | *“I believe that awareness should begin on social media to promote exercise in menopause. Saudi women in this demographic tend to follow celebrities focused on fashion and food, but it’s rare to see much emphasis on health and fitness” Stakeholder 20, Fitness trainer,* WS5 | Social opportunity; Reflective motivation | Social Influences; Reinforcement Beliefs about Consequences | Environmental Restructuring;  Social Support; Modelling; Persuasion | Mass Media and Community Action | **Social opportunity** → via *social norm reshaping* and *visibility enhancement* (MoA), partnerships with influencers, religious leaders, and community organisations can help normalise menopause PA by embedding health messages into trusted social spaces  **Reflective motivation** → via credible role modelling and stigma reduction (MoA), respected figures promoting PA reinforce positive attitudes and address social norms that currently discourage midlife women's participation | **Psychological capability →** via public education, campaigns may embed light educational content (e.g., myth-busting posts, practical tips), though this is less central  **Physical opportunity** → via community resource partnerships and facility access (MoA), e.g., collaborations with gyms, community centers, or workplace wellness programs may provide alternative exercise venues | • Saudi women follow and trust social media influencers for health advice • Religious and community leaders will authentically support PA messaging • Celebrity /influencer endorsement translates to behaviour change rather than just awareness |

Abbreviations: MoA= mechanism of action; HCPs= healthcare professionals
